# A versatile and efficient approach for the synthesis of chiral 1,3-nitroamines and 1,3-diamines via conjugate addition to new (*S*,*E*)-γ-aminated nitroalkenes derived from L-α-amino acids

**DOI:** 10.3762/bjoc.9.95

**Published:** 2013-04-30

**Authors:** Vera Lúcia Patrocinio Pereira, André Luiz da Silva Moura, Daniel Pais Pires Vieira, Leandro Lara de Carvalho, Eliz Regina Bueno Torres, Jeronimo da Silva Costa

**Affiliations:** 1 Núcleo de Pesquisas de Produtos Naturais, Laboratório de Síntese Estereosseletiva de Substâncias Bioativas, Universidade Federal do Rio de Janeiro, 21941-902, Rio de Janeiro, Brazil; 2 Instituto Federal de Educação, Ciência e Tecnologia do Rio de Janeiro, 26530-060, Nilópolis, RJ, Brazil

**Keywords:** amino alcohol, amino aldehyde, azide addition, Baylis–Hillman reaction, cyanide addition, Michael addition

## Abstract

New chiral (*S,E*)-γ-*N*,*N*-dibenzylated nitroalkenes **2a–c** were synthesized from natural L-(α)-amino acids in five steps with overall yields of 68–88%. The conjugate addition of hydride, methoxide, nitronate and azide nucleophiles to **2a–c** led to the corresponding chiral 1,3-nitroamines in 74–90% yield. The conjugate addition of cyanide anion to **2a**,**b** was followed by HNO_2_ elimination affording chiral aminated acrylonitriles (73–98%). On the other hand, the azide anion reacted with **2a**, in acetonitrile, via a [3 + 2]-cycloaddition in which HNO_2_ was lost, providing the corresponding 1,2,3-triazole derivative. Direct reduction of 1,3-nitroamine derivatives **9a**,**b** produced the corresponding 1,3-diamines in good yields.

## Introduction

Nitroalkenes constitute a class of organic compounds that present exceptional versatility in organic synthesis [1–4]. They are reactive in Michael reactions with a wide variety of nucleophiles [5–10], in Friedel–Crafts alkylations [11–14], and Baylis–Hillman reactions [15–18]. Furthermore, nitroalkenes can react as dipolarophiles in [3 + 2] cycloadditions [19–22], as dienophiles in [4 + 2] cycloadditions [23–26], and as heterodienes in hetero-Diels–Alder reactions [27–31] and even participate in cross-coupling reactions [32]. Due to this notable reactivity, obtaining new chiral and achiral nitroalkenes is of great importance in synthetic organic chemistry. Achiral nitroalkenes, specifically the chemically stable β-*trans*-nitrostyrene and its phenyl-substituted analogues, besides the heteroaromatic nitroalkenes, have been employed widely in highly enantioselective syntheses promoted by chiral metallic catalysts [33–37] and most recently by a great number of different chiral organocatalysts [38–41] or combinations of both [42–44]. Nonetheless, the use of chiral nitroalkenes bearing a stereogenic center in the γ-position in nonracemic diastereoselective syntheses ("chiron" approach [45]) is less frequent in the literature. Notably, chiral nitroalkenes derived from carbohydrates [46–50] have been most often utilized as "chirons" in diastereoselective syntheses. Most nitroalkenes are synthesized from a nitroaldol product followed by elimination of the activated hydroxy group in the sulfonate or acetate form [51] although various routes have often been applied [52–54]. On the other hand, chiral nitroamines [55–56] are important intermediates directly transformed, via reduction of the nitro group, into diamines [57] or via a Nef reaction in amino acids [58]. Specifically, chiral 1,2-nitroamines have been mainly accessed by direct nucleophilic addition of nitronate anions to imine derivatives by the use of chiral metal and organocatalysts [55–56]. In contrast, chiral 1,3-nitroamines, despite constituting direct precursors of chiral 1,3-diamines, are scarcely synthesized by conjugate addition of nitroalkanes to nitroalkenes. Chiral 1,3-diamines present extraordinary opportunities in enantioselective synthesis as chiral auxiliaries, chiral catalysts, chiral reagents and chiral ligands [59–60]. In our continuing interest in synthesizing new chiral nonracemic nitro compounds [61–68] by a "chiron" approach [45], we intended to develop a route for obtaining γ-nitrogenated chiral nonracemic nitroalkenes **2a–c** from L-leucine (**1a**), L-phenylalanine (**1b**) and L-alanine (**1c**), respectively, and investigate their reactivities in conjugate additions with different nucleophiles as a way of yielding chiral nonracemic 1,3-nitroamine and its corresponding 1,3-diamine derivatives.

## Results and discussion

**Scheme 1:**
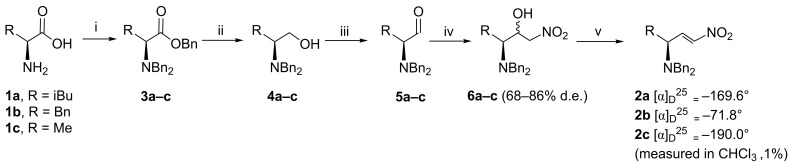
Reagents and conditions: (i) K_2_CO_3_, EtOH/H_2_O 5:1, BnBr, 100 °C, 12 h (90–99%). (ii) (a) LiAlH_4_, THF dry, 100 °C, 8 h. (b) NaOH_aq_ (87–95%). (iii) (a) (COCl)_2_, CH_2_Cl_2_, DMSO, −78 °C, 30 min, then TEA, −78 °C to rt (96–99%). (iv) TBAF·3H_2_O (0.2 equiv), CH_3_NO_2_, THF_dry_, rt, 4 h (91–95%). (v) (a) MsCl, CH_2_Cl_2_, −78 °C, then TEA 60 min, rt (95–99 %).

The preparation of **2a–c** (Scheme 1) was initiated with the synthesis of Reetz’s α-aminoaldehydes [69–70]. In this manner, the α-amino acids **1a–c** were perbenzylated using a slight modification in the experimental procedures described by Beaulieu [71] and Warren [72] leading to tribenzylated esters **3a–c** in 90–99% isolated yield. Next, reduction of **3a–c** with LiAlH_4_ provided the desired alcohols **4a–c** (87–95% yield), which were oxidized under Swern conditions to aldehydes **5a–c** in 96–99% yield. These were submitted to a Henry reaction, according to the modified conditions developed by Hanessian [73]. Thus, **5a–c** were reacted with nitromethane, at room temperature (instead of 0 °C) in the presence of 0.2 equivalents of TBAF·3H_2_O (instead of 1 equivalent) as base, furnishing an *anti*:*syn*-nitroalcohol mixture **6a–c** in 91–95% yield and 68–86% d.e. The activation of the hydroxy groups of **6a–c** by transformation into the corresponding mesylates was followed by smooth elimination at −78 °C, leading to the desired nitroalkenes **2a–c** in 95–99% yield, as single *E*-diastereoisomers. It is worth mentioning that the nitroalkenes **2a–c** are obtained in high chemical and stereochemical purity and can be stored in a freezer or even at room temperature for several months without decomposition occurring.

**Figure 1:**
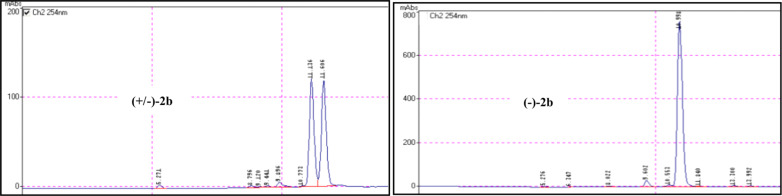
Chiral HPLC analysis: Chiralpak AD-H; *n*-hexane/2-propanol 99:1 (0.6 mL/min), *T* = 25 °C; UV–vis detection at λ = 254 nm; retention times: (+/−)**-2** (11.13 and 11.60 min) and (−)**-2b** (10.99 min).

In order to verify the enantiomeric purity of the new nitroalkenes **2a–c** and to confirm their stereochemical stability, a chiral HPLC analysis was conducted. Racemic (+/−)**-2b** was synthesized from (D/L)-phenylalanine by the same route employed for (−)-**2b** and utilized as standard (Figure 1). The racemic nitroalkene (+/−)-**2b** showed a very good separation factor in the chromatograph chiral column used. Figure 1 shows that (−)-**2b** prepared from L-phenylalanine was enantiomerically pure (enantiomeric excess > 99%).

HPLC analysis showed that the stereochemical integrity of **2** was preserved throughout the synthetic process. Next, the reactivity of the new chiral nitroalkenes was investigated with respect to the conjugate addition of hydride, nitronate, methoxide, cyanide, and azide anions in addition to benzylamine aiming to obtain directly 1,3-nitroamine derivatives. All the experiments were carried out at room temperature and the results are summarized in Table 1.

**Table 1:** Reactivity of **2a–c** with different nucleophiles.

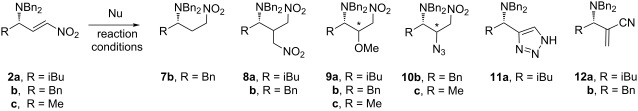						

entry	nitroalkene	Nu	base/solvent/time (h)	adduct	yield (%)	anti/syn^a^

1	**2b**	NaBH_4_	–/CHCl_3_–iPrOH (16:3)/1	**7b**	85	–
2	**2a**	CH_3_NO_2_	DBU 0.3 equiv/CH_3_CN/12	**8a**	86	–
3	**2b**	CH_3_NO_2_	TBAF·3H_2_O/THF/8	**8b**	80	–
4	**2a**	LiOMe^b^	–/MeOH/0.5	**9a**	90	94:6
5	**2b**	LiOMe^b^	–/THF/0.5	**9b**	85	94:6
6	**2a**	LiOMe^b^	–/MeOH/24	**9a**	90	50:50
7	**2b**	LiOMe^b^	–/MeOH/20	**9b**	70	57:43
8	**2c**	LiOMe^b^	–/MeOH/12	**9c**	77	69:31
9	**2a**	TMSCN	TBAF·3H_2_O/CH_3_CN/6	**12a**	98	–
10	**2b**	TMSCN	TBAF·3H_2_O/CH_3_CN/12	**12b**	73	–
11	**2b**	TMSN_3_	TBAF·3H_2_O/THF/16	**10b**	76	86:14
12	**2c**	TMSN_3_	TBAF·3H_2_O/THF/20	**10c**	73	74:26
13	**2a**	TMSN_3_	TBAF·3H_2_O/CH_3_CN/16	**11a**	74	–
14	**2a**	BnNH_2_ excess	–/–/48	–^c^	–	–
15	**2b**	BnNH_2_	DBU 0.5 equiv/CH_3_CN/8	–^c^	–	–

^a^Diastereoisomeric ratio. ^b^Solution 1 M in MeOH. ^c^No reaction observed.

The selective conjugate addition of hydride anion to **2b** produced the chiral 1,3-nitroamine **7b** in 85% yield [74] (Table 1, entry 1). Similarly, the addition of nitromethane to **2a**,**b** led to the corresponding 2-nitromethyl-1,3-nitroamine derivatives **8a**,**b** in good yields (Table 1, entries 2 and 3). Other nitronate anions also reacted adequately. On the other hand, a literature search showed that the addition of an oxygenated nucleophile to nitroalkenes has been scarcely studied due to the low reactivity of neutral nucleophiles associated with the reaction reversibility [46,75–76]. Addition of methoxide anion to **2a–c** led to the corresponding chiral 2-methoxy-1,3*-*nitroamine derivatives **9a–c** in 70–90% yield (Table 1, entries 4–8). A high d.r. was obtained when shorter reaction times were employed (Table 1, entries 4 and 5). On the other hand, a severe decrease in the diastereoselectivity occurred with longer durations, presumably due to an equilibration by a retro-conjugate addition [77] (Table 1, entries 6–8). Next, the conjugate cyanide addition to **2a**,**b** promoted by TMSCN 1 equiv/TBAF·3H_2_O 1 equiv led to **12a**,**b** in 98 and 73% respectively, instead of 2-cyano-1,3*-*nitroamines derivatives (Table 1, entries 9 and 10). Products **12a**,**b** were likely formed from the conjugate addition of cyanide to **2a**,**b** followed by HNO_2_ elimination. It is worthy of note that the Michael addition of “HCN” equivalent to nitroalkenes is rare in the literature [78–83]. Paulsen [78] and Sakakibara [79] were the first authors to report the cyanide addition to α,β-unsaturated nitroalkenes. In agreement with our results, Sakakibara [79] and Spanevello [80] observed a total or partial HNO_2_ elimination from vicinal nitronitrile adducts. This behavior was not observed in other findings reported [78,81–83] and the corresponding vicinal nitronitriles were the sole products. It is important to note that **12a**,**b** can in theory also be obtained by an aza-Baylis–Hillman reaction [15–18,68]. Thus, this cyanide Michael addition to **2a**,**b** constitutes a synthetic alternative to the cases where N-protected aza-Baylis–Hillman adducts need to be obtained. Interestingly, the addition of “HN_3_” promoted by TMSN_3_ 1 equiv/TBAF·3H_2_O 1 equiv furnished either the 2-azido-1,3-nitroamine-derivatives **10b**,**c** (Table 1, entries 11 and 12) or the triazole derivative **11a** (Table 1, entry 13) when THF or CH_3_CN were used as solvents, respectively. The formation of chiral 1,2,3-triazole derivative **11a** was proposed to be by way of a [3 + 2]-cycloaddition with the azide group acting as 1,3-dipolarophile, followed by HNO_2_ elimination leading to aromatization. There are a few reports in the literature where the azide anion is added to α,β-unsaturated nitroalkenes [8,19–22] forming 1,4-adducts [8] or 1,2,3-triazole derivatives [31–33]. Apparently, the reaction course depends on the temperature and concentration of the azide reagent used. In our case, we observed a dependence on the nature of the solvent. However, an influence of the structure of the nitroalkenes has not been independently investigated. Further experiments must be performed for a better understanding of the reaction course. On the other hand, the neat benzylamine addition, or in the presence of DBU (0.5 equiv) as promoter [84], to **2a**,**b** was unsuccessful (Table 1, entries 14 and 15). This nonreactivity can be explained on the basis of the high tendency toward reversibility presented in conjugate additions of amines to reactive nitroalkenes [15,85–86]. As a way of testing the stereochemical stability of the synthesized nitroalkenes in reaction media, **2b** was recovered before the reaction was completed (Table 1, entry 3) and the magnitude of the optical rotation of **2b** was measured. No loss in enantiomeric purity of **2b** was observed in the reaction medium essayed.

**Scheme 2:**
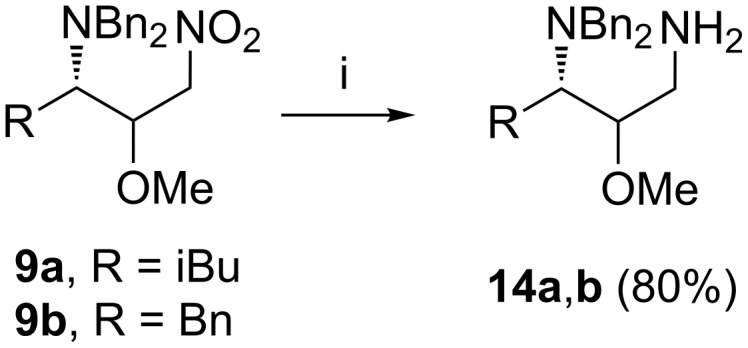
Synthesis of 1,3-diamines **14a**,**b**. (i) NaBH_4_/NiCl_2_·6H_2_O/MeOH/3 h/rt.

Finally, the chiral 1,3-nitroamines **9a**,**b** were easily transformed into the desired chiral 1,3-diamines **14a**,**b** (80% yield) by treatment with NaBH_4_, NiCl_2_·6H_2_O in MeOH [87] (Scheme 2).

## Conclusion

An efficient, reproducible and versatile approach for the synthesis of chiral nonracemic 2-substituted-1,3-nitroamines was developed. The high chemical and stereochemical stability of the new chiral nitroalkenes **2a–c** and the variety of 1,3-nitroamines synthesized provide robustness and generality to the methodology. Since the nitroamines **9a**,**b** were efficiently reduced to the corresponding chiral 2-substituted-1,3-diamines **14a**,**b**, the present route constitutes a versatile access to these important classes of substances in high yields.

## Supporting Information

File 1 General information, experimental procedure and spectroscopic data of **3a–c**, **6a–c**, **2a–c, 7b, 8a, 8b, 11a, 10b–c, 9a–b, 12a, 14a,b**.

File 2 NMR, IR and MS spectra.
