# Supplementary material for: A versatile and efficient approach for the synthesis of chiral 1,3-nitroamines and 1,3-diamines via conjugate addition to new (S,E)-γ-aminated nitroalkenes derived from L-α-amino acids
Source: Beilstein J Org Chem. 2013 Apr 30;9:832–7. doi: 10.3762/bjoc.9.95 (PMC3678503; doi:10.3762/bjoc.9.95)
Supplement: File 1 — General information, experimental procedure and spectroscopic data of 3a–c, 6a–c, 2a–c, 7b, 8a, 8b, 11a, 10b–c, 9a–b, 12a, 14a,b. [file Beilstein_J_Org_Chem-09-832-s001.pdf]

# Supporting Information

for

# A versatile and efficient approach for the synthesis of chiral 1,3-nitroamines and 1,3-diamines via conjugate addition to new (*S*,*E*)-γ-aminated nitroalkenes derived from L-α-amino acids

Vera Lúcia Patrocinio Pereira[1,§,*], André Luiz da Silva Moura[1], Daniel Pais Pires Vieira[1], Leandro Lara de Carvalho[1], Eliz Regina Bueno Torres[1] and Jeronimo da Silva Costa[2]

Address: [1]Núcleo de Pesquisas de Produtos Naturais, Laboratório de Síntese Estereosseletiva de Substâncias Bioativas, Universidade Federal do Rio de Janeiro, 21941-902, Rio de Janeiro, Brazil and [2]Instituto Federal de Educação, Ciência e Tecnologia do Rio de Janeiro, 26530-060, Nilópolis, RJ, Brazil

Email: Vera Lúcia Patrocinio Pereira - patrocinio@nppn.ufrj.br

* Corresponding author

[§]Tel.: +55 21 2562 6792; Fax: +55 21 2562 6512

## General information, experimental procedure and spectroscopic data of **3a–c**, **6a–c**, **2a–c, 7b, 8a, 8b, 11a, 10b–c, 9a–b, 12a, 14a,b**

## General information

TBAF·3$H_2O$ solid, $TMSN_3$, TMSCN, Amberlyst-A21 and nitromethane are commercially available (Aldrich, Acros or Merck) and were used as received. Triethylamine, $CH_3CN$ and $CH_2Cl_2$ were dried over $CaH_2$. THF was dried according to literature procedures [1]. $^1$H NMR and $^{13}$C NMR spectra were recorded on Varian MR-400 (400 MHz) or Gemini-200 (200 MHz) with TMS as an internal reference. Chemical shifts (δ) are reported in ppm. The IR spectra were recorded on a Shimadzu IR-Prestige-21 (FTIR) and only the principal bands are reported. The melting points are uncorrected and were determined on a Thomas Hoover apparatus. Optical rotations were recorded at 20 °C using a Jasco P-2000 (PTC-203) polarimeter. GC-mass analyses were performed on a Shimadzu GC/MS-QP 500 spectrometer. Chromatography was performed with an HPLC system, consisting of a Shimadzu LC-10AD pump and a Shimadzu SPD-M10A variable-wavelength UV–vis detector with the detection set at 254 nm. Daicel Chiralpack AD-H chiral column was eluted with a hexane/2-propanol (99:1) mixture (0.6 mL/min). HRMS (ESI) experiments were performed in positive mode using a Bruker micrOTOF II.

## Synthesis of tribenzylated amino esters 3a–c [2-4]. Typical procedure:

### (*S*)-Benzyl 2-(dibenzylamino)-4-methylpentanoate (3a)

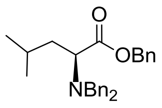


**3a**

L-leucine (5.0 g, 38.1 mmol), $K_2CO_3$ (15.8 g, 114.3 mmol), EtOH (67 mL) and $H_2O$ (13 mL) were added to a two-neck round-bottom flask, and the system was heated under reflux with magnetic stirring until complete dissolutions was obtained. Then, BnBr

(19.5 g, 114.3 mmol) was added dropwise and heating under reflux was maintained overnight (12 h). After cooling, $H_2O$ (20 mL) and brine (30 mL) were added and the aqueous phase was extracted with EtOAc (3 × 30 mL). The organic layers were combined, dried over $Na_2SO_4$, filtered and concentrated under reduced pressure furnishing 15.3 g of **3a** (98% yield) as a fluid colorless oil. No further purification was necessary. (Thin layer chromatography, $R_f$ = 0.8, EtOAc/Hex 1:4).

$[\alpha]_D^{25} = -9.5$ (*c* 0.82; $CHCl_3$).

IR (film): *ν* 2954, 2867, 2000, 1800, 1731, 1602, 1494, 1454, 1367, 1211, 1172, 1074, 1029, 746, 698 $cm^{-1}$.

$^1H$ NMR (200 MHz, $CDCl_3$): δ 7.3 (15H, m), 5.3 (1H, d, *J* = 12.0 Hz), 5.1 (1H, q, *J* = 12.5 Hz), 3.9 (2H, d, *J* = 13.6 Hz), 3.5 (2H, d, *J* = 13.9 Hz), 3.48-3.35 (1H, m), 1.85-1.62 (2H, m), 1.58-1.40 (1H, m), 0.80 (3H, d, *J* = 6.6 Hz), 0.60 (3H, d, *J* = 6.2 Hz).

$^{13}C$ NMR (50 MHz, $CDCl_3$): δ 173.2, 139.6, 136.1, 128.9, 128.1, 126.9, 65.9, 58.8, 54.4, 38.6, 24.4, 23.2, 21.5.

**Synthesis of nitroalcohols 6a–c [5]. Typical procedure:**

**(3*S*)-3-(*N*,*N*-Dibenzylamino)-5-methyl-1-nitrohexan-2-ol (6a**).

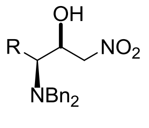


**6a**, R = iBu
**6b**, R = Bn
**6c**, R = Me

To a round-bottom flask at room temperature, dry THF [1] (5.0 mL), TBAF•$3H_2O$ (0.252 g, 0.80 mmol) and $CH_3NO_2$ (260 μL, 4.8 mmol) was added sequentially. After 10 min a solution of **5a** (1.2 g, 4.0 mmol in 5.0 mL of THF) was added, and the reaction was maintained under constant stirring for 4 h. The reaction mixture was diluted with

$H_2O$ (20.0 mL) and extracted with EtOAc (3 × 20.0 mL). The combined organic layers were dried with $Na_2SO_4$, filtered and concentrated under reduced pressure affording 1.45 g of **6a**, in 98% yield, as a viscous yellow-brown oil constituted by a diastereomer mixture (84:16). Thin-layer chromatograph ($R_f$ = 0.34 and 0.26, EtOAc/Hex 10:90).

IR (film): ν 3565, 3440, 2956, 2931, 2867, 2000-1800, 1602, 1556, 1494, 1454, 1420, 1378, 1205, 1141, 1074, 1027, 750, 700 $cm^{-1}$.

$^1$H NMR (200 MHz, $CDCl_3$): δ 7.40-7.17 (10H, m), 5.27 (1H, s), 4.55-4.35 (1H, m), 4.30-4.10 (1H, m), 3.90 (2H, d, *J* = 13.2 Hz), 3.75-3.45 (1H, m), 3.40 (2H, d, *J* = 13.2 Hz), 2.60 (1H, m), 1.60 (2H, m), 1.50 (1H, m), 1.00 (3H, d, *J* = 5.9 Hz), 0.90 (3H, d, *J* = 5.5 Hz).

$^{13}$C NMR (50 MHz, $CDCl_3$): δ 138.9, 128.9, 128.5, 127.4, 80.1, 69.5, 57.2, 54.4, 34.1, 25.9, 23.5, 22.4.

**Synthesis of nitroalkenes 2a–c. Typical procedure:**

**(*S,E*)-*N,N*-Dibenzyl-5-methyl-1-nitrohex-1-en-3-amine (2a).**

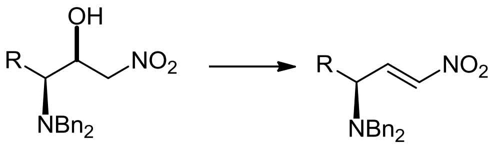


**6a**, R = iBu
**6b**, R = Bn
**6c**, R = Me

**2a**–c

To a round-bottom flask, at −78 °C, under argon atmosphere were added MsCl (0.22 mL, 2.8 mmol) and $CH_2Cl_2$ (5.0 mL) under constant stirring. Next, a solution of

**6a** (0.5 g, 1.40 mmol in 5 mL of $CH_2Cl_2$ or THF) as a mixture of diastereomers was added, and the system was reacted for 20 min. Then, TEA (1.37 mL, 9.8 mmol) was added and stirring was continued for 1 h. The reaction was diluted with $CH_2Cl_2$ (30.0 mL) and washed with HCl 10% (2 × 20 mL) and $H_2O$ (30.0 mL). The combined organic layers were dried over $Na_2SO_4$, filtered and concentrated under reduced pressure affording 0.48 g of **2a** (99% yield), as a yellow-brown oil in satisfactory purity. **Note:** Prolonged contact of the nitroalkene on silica gel via an eventual purification through a chromatographic column causes severe product loss.

$[\alpha]_D^{25}$ = −169.6 (*c* 1.0, $CHCl_3$).

IR (Film): ν 2956, 2931, 2869, 1641, 1602, 1556, 1525, 1494, 1454, 1369, 1351, 1205, 1172, 1153, 1074, 1027, 968, 748, 700 $cm^{-1}$.

$^1$H NMR (400 MHz, $CDCl_3$): δ 7.37-7.28 (11H, m), 7.0 (1H, d, *J* = 13.4 Hz), 3.83 (2H, d, *J* = 13.7 Hz), 3.39 (2H, d, *J* = 13.7 Hz), 3.41-3.36 (1H, m), 1.78-1.69 (2H, m), 1.37-1.29 (1H, m), 0.81 (3H, d, *J* = 6.4 Hz), 0.72 (3H, d, *J* = 6.2 Hz).

$^{13}$C NMR (100 MHz, $CDCl_3$): δ 141.0, 140.5, 138.9, 128.6, 128.4, 127.2, 53.8, 53.7, 40.1, 24.4, 22.6, 22.3.

GC/MS (70 eV) *m/z* 281 ($M^{+\bullet}$ − $CH_2CH(CH_3)_2$, 30), 144, 115, 104, 91, 77, 65, 43.

HRMS-ESI (*m/z*) calcd for $C_{21}H_{27}N_2O_2$ $[M + H]^+$ 339.2067, found 339.2095.

**(*S*,*E*)-*N*,*N*-Dibenzyl-4-nitro-1-phenylbut-3-en-2-amine (2b).**

Yellow crystalline solid.

$[\alpha]_D^{25}$ = −71.8 (*c* 1.0, $CHCl_3$); mp = 59–61 °C.

IR (KBr): ν 3061–3085, 2928, 2845, 1670, 1526, 1350 $cm^{-1}$.

$^{1}$H NMR (400 MHz, $CDCl_3$): δ 7.23-7.32 (14H, m), 7.02 (2H, d, *J* = 7.5 Hz), 6.91 (1H, d, *J* = 13.4 Hz), 3.85 (2H, d, *J* = 13.8 Hz), 3.57 (2H, d, *J* = 13.8 Hz), 3.66 (1H, m), 3.19 (1H, dd, *J* = 13.6, 6.39 Hz), 2.82 (1H, dd, *J* = 13.6, 8.6 Hz).

$^{13}$C NMR (100 MHz, $CDCl_3$): δ 140.8, 140.2, 138.6, 137.4, 129.3-126.7, 58.0, 53.8, 36.7.

GC/MS (70 eV) *m/z* 300 ($M^{+\bullet}$ − HC=$CHNO_2$, 20), 281, 181, 144, 128, 115, 105, 91, 77, 65, 51.

HRMS-ESI (*m/z*) calcd for $C_{24}H_{25}N_2O_2$ $[M + H]^+$ 373.1911 found 373.1925.

**(*S*,*E*)-*N*,*N*-Dibenzyl-4-nitrobut-3-en-2-amine (2c)**

Yellow-brown oil; $[\alpha]_D^{25}$ = −190.0 (*c* 1.0, $CHCl_3$).

IR (Film): *v* 3062–3085, 2973, 2933, 2880, 2837, 1525, 1351, 1028 $cm^{-1}$.

$^{1}$H NMR (400 MHz, $CDCl_3$): δ 7.22-7.38 (11H, m), 7.00 (1H, d, *J* = 13.5 Hz), 3.61 (5H, m), 1.28 (3H, d, *J* = 6.5 Hz).

$^{13}$C NMR (100 MHz, $CDCl_3$): δ 143.9, 140.0, 139.0, 128.4–127.2, 53.8, 51.4, 13.9; GC/MS (70 eV): *m/z* 296 ($M^{+\bullet}$, 1%), 281, 279, 205, 144, 132, 104, 91, 77, 65, 51.

***N*,*N*-Dibenzyl-4-nitro-1-phenylbutan-2-amine (7b)**

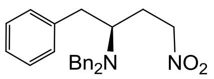


**7b**

To a stirred solution of **2b** (97.1 mg, 0.260 mmol) in $CHC1_3$ (4.3 mL) and 2-propanol (0.80 mL) was added $NaBH_4$ (40 mg, 4.0 mmol) in 10 mg portions over a period of 60 min at 25 °C. Excess $NaBH_4$ was decomposed with HCl 10%, and the mixture was

extracted with $CH_2C1_2$. The combined organic layers were washed with brine, dried ($Na_2SO_4$ in the presence of $NaHCO_3$) and evaporated under reduced pressure to give a crystalline white solid **7b** (83.3 mg, 0.222 mmol) in 85% yield.

$[\alpha]_D^{25}$ = +28.6 (*c* 1.38, $CHCl_3$).

$^1H$ NMR (400 MHz, $CDCl_3$) δ 6.99-7.27 (15 H, m), 4.43 (1H, ddd, *J* = 13.6, 8.7, 5.4 Hz), 3.99 (1H, m), 3.82 (2H, d, *J* = 13.4 Hz), 3.40 (2H, d, *J* = 13.4 Hz), 3.17 (1H, dd, *J* = 13.0, 2.6 Hz), 2.77 (1H, m), 2.36 (1H, dd, *J* = 12.3, 11.1 Hz), 2.11 (1H, m), 1.86 (1H, m).

$^{13}C$ NMR (100 MHz, $CDCl_3$) δ 139.2, 129.1, 128.8, 128.6, 128.5, 127.3, 126.3, 73.5, 57.5, 53.4, 34.1, 28.0.

**(*S*)-*N*,*N*-Dibenzyl-5-methyl-1-nitro-2-(nitromethyl)-hexan-3-amine (8a).**

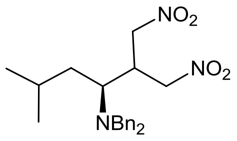


**8a**

To a round-bottom flask, at room temperature, MeCN (1.0 mL), $CH_3NO_2$ (0.095 mL, 1.78 mmol, 2.0 equiv) and DBU (0.067 mL, 0.45 mmol, 0.5 equiv) were added sequentially. After 10 min a solution of **2a** (0.3 g, 0.89 mmol, 1.0 equiv) in dry MeCN (1 mL) was added and reaction mixture was stirred for 24 h at room temperature. Then, the reaction mixture was diluted in EtOAc (30 mL) and extracted with $H_2O$ (3 × 20 mL). The organic layers were dried over $Na_2SO_4$, filtered and concentrated under reduced pressure furnishing 0.301 g of **8a** (86% yield) as a viscous brown-red oil.

$[\alpha]_D^{25}$ = +9.8 (*c* 1.05, $CHCl_3$).

IR (Film): ν 2956, 2929, 2867, 2000, 1800, 1602, 1558, 1540, 1494, 1454, 1369, 1346, 1207, 1145, 1074, 1027, 748, 700 $cm^{-1}$.

$^{1}$H NMR (200 MHz, $CDCl_3$): δ 7.40-7.00 (10H, br s), 4.70 (1H, m), 4.10 (1H, m), 3.85 (2H, d, $J$ = 13.0 Hz), 3.60 (1H, m), 3.40 (2H, d, $J$ = 14.0 Hz), 3.26-3.10 (1H, m), 2.08-2.01 (1H, bs), 1.69-1.57 (1H, bb), 1.52 (3H, s), 1.30-1.16 (1H, m), 1.26 (3H, s), 0.95 (3H, d, $J$ = 7.8 Hz), 0.85 (3H, d, $J$ = 7.6 Hz).

$^{13}$C NMR (50.4 MHz, $CDCl_3$): δ 139.1, 128.9, 128.3, 127.2, 89.9, 73.5, 53.9, 52.8, 43.2, 38.0, 25.1, 24.4, 24.2, 23.7, 20.6.

**(*S*)-*N*,*N*-Dibenzyl-4-nitro-3-(nitromethyl)-1-phenylbutan-2-amine (8b).**

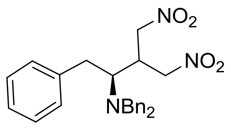


**8b**

To a round-bottom flask, at room temperature, a solution of TBAF•$3H_2O$ (0.0945 g, 0.3 mmol, 0.3 equiv) in THF (2 mL) and $CH_3NO_2$ (0.023 mL, 0.44 mmol, 1.3 equiv) were added and stirred for 10 minutes. Next, nitroalkene **2b** (0.12 g, 0.335 mmol, 1.0 equiv) dissolved in 1.0 mL of THF was added and the stirring was continued for 4.0 h. Then, the reaction mixture was diluted in EtOAc (30 mL) and extracted with $H_2O$ (3 × 20 mL). The organic layers were dried over $Na_2SO_4$, filtered and concentrated under reduced pressure. Purification on silica gel chromatographic column, eluted with hexane/EtOAc 90:10, produced 0.112 g (80% yield) of **8b** as a viscous yellow oil.

$[\alpha]_D^{25}$ = +16.7 (*c* 0.82, $CHCl_3$).

IR (Film): ν 3085, 3062, 3028, 2924, 2851, 1552, 1495, 1454, 1376, 1263, 1122, 1028, 969, 747, 699 $cm^{-1}$.

$^{1}H$ NMR (400 MHz, $CDCl_3$): δ 7.17-7.40 (15H, m), 4.80 (2H, dd, *J* = 14.2, 3.6 Hz), 4.41 (2H, dd, *J* = 14.4, 8.0 Hz), 4.32 (1H, dd, *J* = 13.0, 5.2 Hz), 4.26 (1H, dd, *J* = 14.8, 6.0 Hz), 3.83 (2H, d, *J* = 13.7 Hz), 3.44 (2H, d, *J* = 13.5 Hz), 3.34-3.26 (1H, m), 3.15-3.06 (1H, m).

$^{13}C$ NMR (100 MHz, $CDCl_3$): δ 138.65, 138.26, 129.10, 128.94, 128.70, 127.67, 127.01, 74.26, 74.17, 58.25, 54.43, 39.40, 33.31.

**(*S*)-*N*,*N*-Dibenzyl-3-methyl-1-(1*H*-1,2,3-triazol-4-yl)butan-1-amine (11a) - tautomers mixture.**

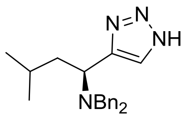


**11a**

To a round-bottom flask, at room temperature and under magnetic stirring, a solution of TBAF·$3H_2O$ (0.139 g, 0.44 mmol, 1.0 equiv) in $CH_3CN$ and $TMSN_3$ (0.071 mL, 0.53 mmol, 1.2 equiv) were added, and stirring was continued for 10 minutes. Then, nitroalkene **2a** (0.44 mmol, 1.0 equiv) dissolved in 1.0 mL of $CH_3CN$ was added, and the reaction was stirred overnight, at room temperature. Next, the solvent was removed under reduced pressure, and the remaining residue was dissolved in $CH_2Cl_2$ (30 mL) and extracted with $H_2O$ (3 × 30 mL). The combined organic layers were dried over $Na_2SO_4$, filtered across column chromatography on silica gel, and concentrated under reduced pressure to furnish 0.107 g of **11a** as a yellow oil (74% yield).

$[\alpha]_D^{25} = -3.0$ (*c* 1.0, $CHCl_3$).

IR (Film): ν 2960, 2933, 2873, 2103, 2000, 1800, 1610, 1556, 1494, 1454, 1378, 1367, 1286, 1207, 1074, 1039, 1027, 962, 748, 700 $cm^{-1}$.

$^{1}$H NMR (200 MHz, $CDCl_3$): δ 7.60 (1H, s), 7.42-7.23 (10H, m), 4.00 (1H, t, *J* = 7.3 Hz), 3.80 (2H, d, *J* = 13.7 Hz), 3.20 (2H, d, *J* = 13.7 Hz), 2.00-1.60 (6H, m), 0.80 (3H, d, *J* = 6.3 Hz), 0.70 (3H, d, *J* = 6.3 Hz).

$^{13}$C NMR (50.4 MHz, $CDCl_3$): δ 161.8, 140.1, 139.0, 133.5, 132.3, 128.8, 128.0, 126.5, 58.5, 55.5, 52.4, 51.2, 24.4, 23.8, 22.8, 22.1, 19.5, 13.5.

**Typical procedure for nitro-azide derivatives 10b,c;**

**(2*S*)-3-Azido-*N,N*-dibenzyl-4-nitro-1-phenylbutan-2-amine (10b).**

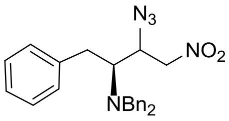


**10b**

To a stirred solution of TBAF·3$H_2O$ (0.189 g, 0.6 mmol) in anhydrous THF (5 mL) at room temperature was added trimethylsilyl azide (0.08 mL, 0.6 mmol). Next, the nitroolefin **2b** (0.20 g, 0.54 mmol) dissolved in THF (5 mL) was added and reaction mixture was stirred for 20 hours. Then, the reaction mixture was washed with water (40 mL) and extracted with AcOEt (3 × 10 mL). The combined organic layers were dried over $Na_2SO_4$, filtered and concentrated under reduced pressure. Purification on a silica-gel chromatographic column, eluted with hexane/AcOEt 80:20, furnished 0.169 g (76% yield) of **10b** as a viscous yellow-brown oil consisting of a diastereomeric mixture (86:14).

IR (Film): ν 2132, 2100, 1555, 1377, 1075, 1024, 747, 700 $cm^{-1}$.

$^{1}$H NMR (200 MHz, $CDCl_3$): δ 7.4 – 7.2 (15H, m), 4.75 (2H, m), 4.16 (1H, m), 3.8 (4H, m), 3.4 (1H, d, 13.4Hz), 3.0 (2H, m).

$^{13}$C NMR (50.4 MHz, $CDCl_3$): δ 140.1, 129.1 – 126.5, 77.8, 61.0, 60.6, 55.3, 30.2.

GC/MS (70 eV): *m/z* 197, 120, 106, 91, 65, 51.

**(2*S*)-3-Azido-*N,N*-dibenzyl-4-nitrobutan-2-amine (10c)**

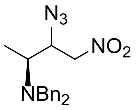


**10c**

Diastereomeric mixture (74:26).

IR (Film): ν = 3086, 3062, 2972, 2930, 2835, 2807, 2131, 2100, 1556, 1494, 1453, 1379, 748, 700 cm$^{-1}$.

$^{1}$H NMR (200 MHz, $CDCl_3$): δ 7.6-7.2 (10H, m), 5.02 (1H, dd, *J* = 13.9, 2.38 Hz), 4.6-4.2 (1H, m), 4.1-3.45 (5H, m), 3.4 (1H, d, *J* = 13.4 Hz), 3.0-2.6 (1H, m), 1.4-1.2 (3H, d, *J* = 6.7 Hz).

**Typical procedure for methoxylated derivatives 9a–c;**

**Synthesis of (2*S*)-*N,N*-dibenzyl-3-methoxy-4-nitro-1-phenylbutan-2-amine (9b),** (Table 1, entry 5).

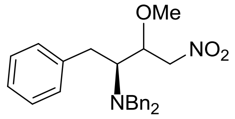


**9b**

To a round-bottom flask at room temperature a solution of **2b** (0.214 g, 0.574 mmol) in dry THF (2.0 mL), under an inert atmosphere, MeOLi/MeOH (0.574 mmol, 0.26 mL, 2.2 mmol/mL) was added, and stirring was continued for 60 min. After, the reaction mixture was diluted with $CH_2Cl_2$ (30 mL), and washed with HCl 5% (10 mL) and $H_2O$

(3 × 15 mL). The organic phase was dried over $Na_2SO_4$, filtered and concentrated under reduced pressure furnishing 0.197 g of **9b** as a yellow crystalline solid in 85 % yield, in satisfactory purity as a mixture of diastereomers (94:6).

$[\alpha]_D^{25}$ = +6.3° (*c* 1.00, $CHCl_3$), mp 102–104 °C.

IR (Film,): ν 3448, 3082, 3026, 2926, 2839, 1550, 1454, 1379, 1136, 1105, 1074, 744, 700 $cm^{-1}$.

$^1$H NMR (400 MHz, $CDCl_3$): δ 7.34-7.00 (15H, m), 4.77 (1H, dd, *J* = 13.5, 7.6 Hz), 4.15 (3H, m), 3.70 (1H, m), 3.41 (2H, d, *J* = 13.7 Hz), 3.31 (3H), 3.16 (1H, dd, *J* = 12.9, 3.5 Hz), 2.99 (1H, m), 2.90 (1H, m).

$^{13}$C NMR (100 MHz, $CDCl_3$): δ 139.8, 139.3, 129.5-126.5, 80.0, 79.1; 61.7, 60.0, 55.6, 29.4.

HRMS-ESI (*m*/*z*) calcd for $C_{25}H_{29}N_2O_3^+$ $[M + H]^+$ 405.2173, found 405.2174.

**(3S)-*N*,*N*-Dibenzyl-2-methoxy-5-methyl-1-nitrohexan-3-amine (9a) (Table 1, entry 4)** mixture of diastereomers (94:6)

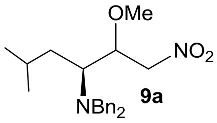


**9a**

$[\alpha]_D^{25}$ = −22.4 (*c* 2.26, $CHCl_3$).

IR (Film): ν 2956, 2933, 2867, 2836, 1602, 1552, 1494, 1454, 1382, 1205, 1159, 1103, 1074, 1027, 750, 700 $cm^{-1}$.

$^1$H NMR (400 MHz, $CDCl_3$): δ 7.34-7.23 (10H, m), 4.80 (1H, dd, *J* = 13.7, 8.2 Hz), 4.15 (1H, dd, *J* = 13.5, 2.9 Hz), 4.00 (2H, d, *J* = 13.3 Hz), 4.00 (1H, m), 3.37 (3H, s),

3.32 (2H, d, $J$ = 13.3 Hz), 2.65 (1H, m), 1.75 (1H, m), 1.65 (1H, m), 1.50 (1H, m), 1.00 (3H, d, $J$ = 6.3 Hz), 0.90 (3H, d, $J$ = 6.3 Hz).

$^{13}$C NMR (100 MHz, $CDCl_3$): δ 140.0, 128.9, 128.3, 127.1, 80.6, 79.2, 60.5, 56.8, 55.2, 32.1, 25.1, 24.0, 22.9.

**(*S*)-*N,N*-3-(Dibenzylamino)-5-methyl-2-methylenehexanenitrile (12a).**

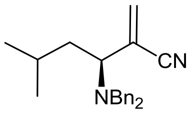


**12a**

To a solution of TBAF•$3H_2O$ (0.139 g, 0.44 mmol, 1.0 equiv) in $CH_3CN$ (3 mL) was added TMSCN (0.071 mL, 0.53 mmol, 1.2 equiv). After 10 minutes **2a** (0.15 g, 0.44 mmol, 1.0 equiv) dissolved in 1.0 mL of $CH_3CN$ was added, and the reaction was stirred for 6 h, at room temperature, under an inert atmosphere. The $CH_3CN$ was removed under reduced pressure and the reaction mixture was dissolved with $CH_2Cl_2$ (30 mL) and extracted with $H_2O$ (2 × 10 mL) and brine (2 × 10 mL). The organic phase was dried over $Na_2SO_4$, filtered across a silica-gel chromatographic column and concentrated under reduced pressure affording **12a** as a brown viscous liquid in 98% yield.

$[\alpha]_D^{25} = -21.9$ ($c$ 1.0, $CHCl_3$).

IR (Film): ν 2956, 2929, 2869, 2217, 2000, 1800, 1602, 1494, 1454, 1369, 1205, 1133, 1074, 1027, 944, 746, 698 cm$^{-1}$.

$^{1}$H NMR (400 MHz, $CDCl_3$): δ 7.45-7.20 (10H, m), 6.12 (1H, s), 5.64 (1H, s), 3.90 (2H, d, *J* = 13.9 Hz), 3.40 (2H, d, *J* = 13.9 Hz), 3.30 (1H, t, *J* = 7.0 Hz), 1.75 (2H, m), 1.60 (1H, m), 0.80 (6H, d, *J* = 7.1 Hz).

$^{13}$C NMR (100 MHz, $CDCl_3$): δ 139.2, 132.8, 128.6, 128.3, 127.1, 122.4, 118.8, 58.4, 53.3, 38.8, 24.5, 22.6, 22.5.

**Typical procedure for diamine derivatives 14a–b.**

**(3S)-*N*$^{3}$,*N*$^{3}$-Dibenzyl-2-methoxy-4-phenylbutane-1,3-diamine (14b)**

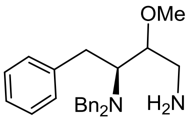


**14b**

To a round-bottom flask at room temperature $NiCl_2 \cdot 6H_2O$ (0.144 g, 0.604 mmol, 2.5 equiv) and $NaBH_4$ (0.274g, 7.26 mmol, ~30 equiv) were added to a stirred solution of **9b** (0.098 g, 0.242 mmol) in MeOH (2.5 mL). The resulting dark suspension was stirred for 3 h. After, the mixture was quenched with water (10 mL) and HCl 10% until the approximately pH 2, and then alkalinized with NaOH solution 1.0 M until approximately pH 9. The product was extracted with AcOEt (3 × 10 mL) and the combined organic layers were washed with brine, dried ($MgSO_4$), filtrated through Celite, and concentrated under reduced pressure to afford **14b** (0.072 g, 80% yield) as a yellow oil.

$[\alpha]_D^{25}$ = +27.6 (*c* 2.60, $CHCl_3$).

IR (Film): ν 2924, 2364, 1650, 1492, 1454, 1101, 746, 700 cm$^{-1}$.

$^{1}$H NMR (400 MHz, $CDCl_3$): δ 7.38-7.15 (15H, m), 4.14 (2H, d, *J* = 13.7 Hz), 3.62 (1H, m), 3.46 (2H, d, *J* = 13.2 Hz), 3.30 (3H, m), 3.14 (1H, dd, *J* = 13.0, 4.2 Hz), 3.00 (1H, m), 2.90 (3H, m), 2.78 (1H, m), 2.73 (1H, dd, *J* = 12.2, 4.4 Hz).

$^{13}$C NMR (100 MHz, $CDCl_3$): δ 140.4, 129.3-125.8, 83.4, 59.6, 58.2, 55.5, 41.2, 29.5.

HRMS-ESI (*m*/*z*) calcd for $C_{25}H_{31}N_2O^+$ $[M + H]^+$ 375.2431, found 375.2442.

***N*$^3$,*N*$^3$-Dibenzyl-2-methoxy-5-methylhexane-1,3-diamine (14a)**

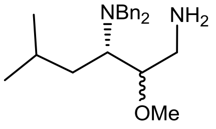


**14a**

IR (Film): ν 2954, 2926, 2366, 2341, 1637, 1261, 1122, 771, 744, 702 cm$^{-1}$.

$^{1}$H NMR (400 MHz, $CDCl_3$): δ 7.38-7.14 (10H, m), 3.92 (2H, d, *J* = 12.0 Hz), 3.43 (2H, d, *J* = 12.0 Hz), 3.34 (3H, s), 3.10 (1H, m), 2.88 (1H, m), 2.81 (1H, m), 2.30 (1H, m), 1.62 (m,1H), 1.52 (1H, m), 1.45 (1H, m), 0.85 (bs, 6H).

HRMS-ESI (*m*/*z*) calcd for $C_{22}H_{33}N_2O^+$ $[M + H]^+$340.2515, found 341.2597

**References:**

1. Simas, A. B. C.; Pereira V. L. P.; Jr-Barreto C. B.; Sales, D. L.; Carvalho, L. L. *Quim. Nova* **2009**, *32,* 2473-2475.

2. Reetz, M. T. *Chem. Rev.* **1999**, *99*, 1121-1162.

3. Beaulieu, P. L.; Wernic, D. *J. Org. Chem.* **1996**, *61*, 3635-3645.

4. O'Brien, P.; Warren, S. *Tetrahedron Lett.* **1996**, *37,* 4271-4274.

5. Hanessian, S., Devasthale, P. V. *Tetrahedron Lett.* **1996**, *37*, 987-990.
