# Supplementary material for: A versatile and efficient approach for the synthesis of chiral 1,3-nitroamines and 1,3-diamines via conjugate addition to new (S,E)-γ-aminated nitroalkenes derived from L-α-amino acids
Source: Beilstein J Org Chem. 2013 Apr 30;9:832–7. doi: 10.3762/bjoc.9.95 (PMC3678503; doi:10.3762/bjoc.9.95)
Supplement: File 2 — NMR, IR and MS spectra. [file Beilstein_J_Org_Chem-09-832-s002.pdf]

Supporting Information

for

A versatile and efficient approach for the synthesis of chiral 1,3-nitroamines and 1,3-diamines via conjugate addition to new (*S*,*E*)-γ-aminated nitroalkenes derived from L-α-amino acids

Vera Lúcia Patrocinio Pereira[1,§,*], André Luiz da Silva Moura[1], Daniel Pais Pires Vieira[1], Leandro Lara de Carvalho[1], Eliz Regina Bueno Torres[1] and Jeronimo da Silva Costa[2]

Address: [1]Núcleo de Pesquisas de Produtos Naturais, Laboratório de Síntese Estereosseletiva de Substâncias Bioativas, Universidade Federal do Rio de Janeiro, 21941-902, Rio de Janeiro, Brazil and [2]Instituto Federal de Educação, Ciência e Tecnologia do Rio de Janeiro, 26530-060, Nilópolis, RJ, Brazil

Email: Vera Lúcia Patrocinio Pereira - patrocinio@nppn.ufrj.br

* Corresponding author

[§]Tel.: +55 21 2562 6792; Fax: +55 21 2562 6512

NMR, IR and MS spectra

Contents:

Copy of ^{1}H NMR spectra of **2a–c**, **8a**, **8b**, **7b**, **9b**, **9a**, **10b**, **11a**, **12a**, **14b**.

Copy of ^{13}C NMR spectra of **11a**, **7b**, **8b**, **9a**, **12a**, **2a**, **2c** and APT of **2b**, **8a**, **9a**, **9b**, **10b**, **14b**.

Copy of IR spectra of **11a**, **12a**, **9a**, **14b**.

Copy of HRMS spectra of **14a**, **14b**, **9b**, **7b**, **2a**, **2b**.

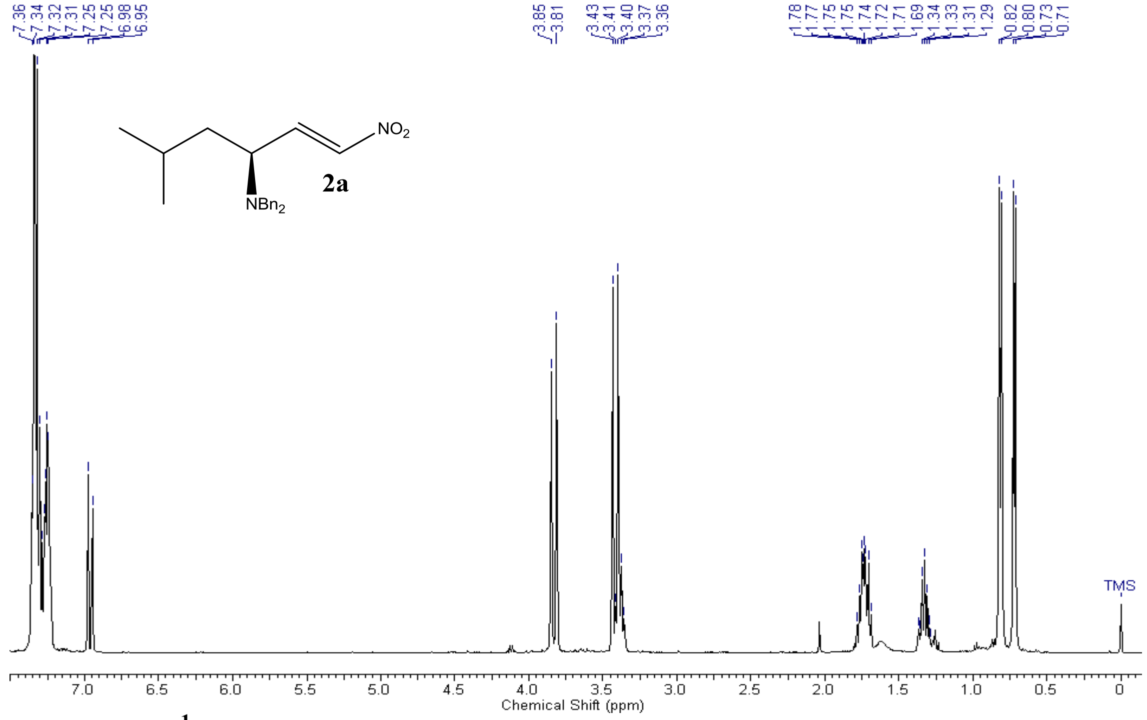

Spectrum 1: ^{1}H NMR (400 MHz, $CDCl_3$) of 2a

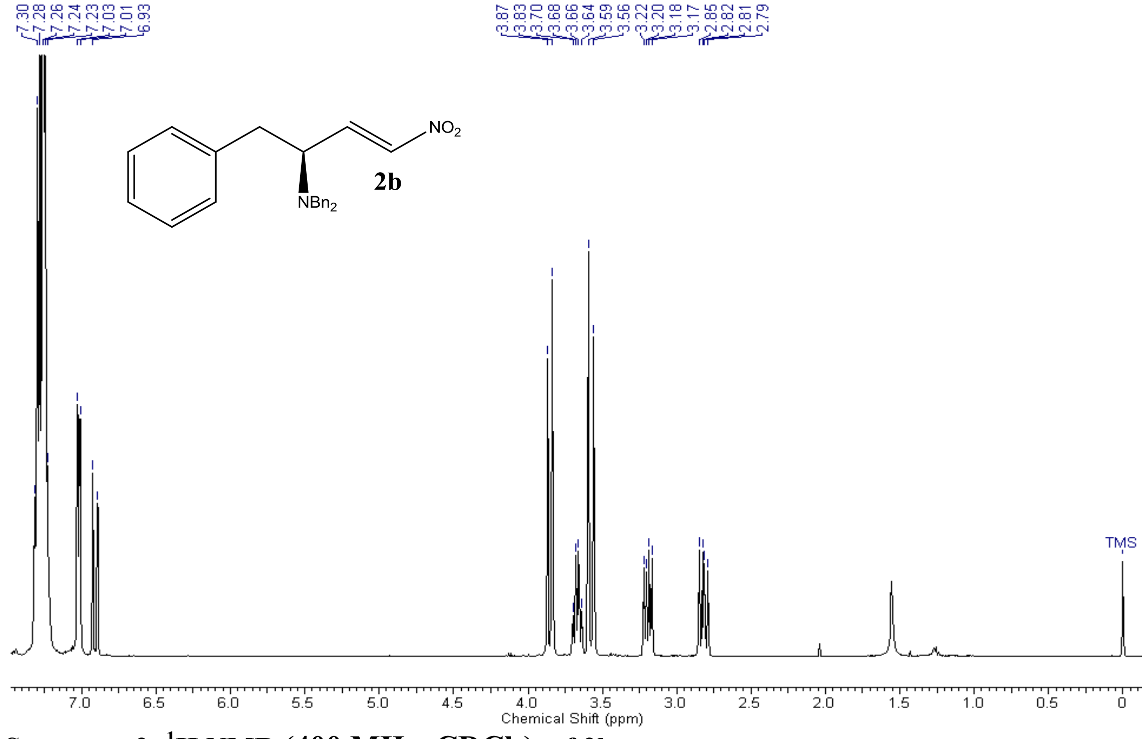

Spectrum 2: ^{1}H NMR (400 MHz, $CDCl_3$) of 2b

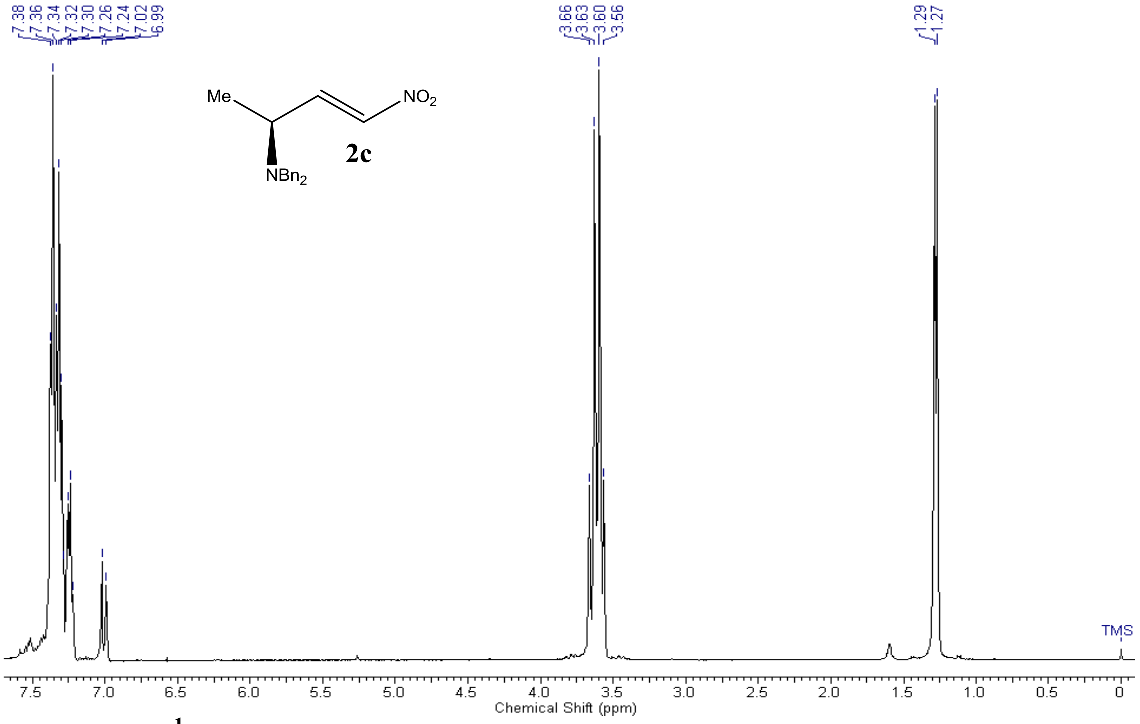

Spectrum 3: ^{1}H NMR (400 MHz, $CDCl_3$) of 2c

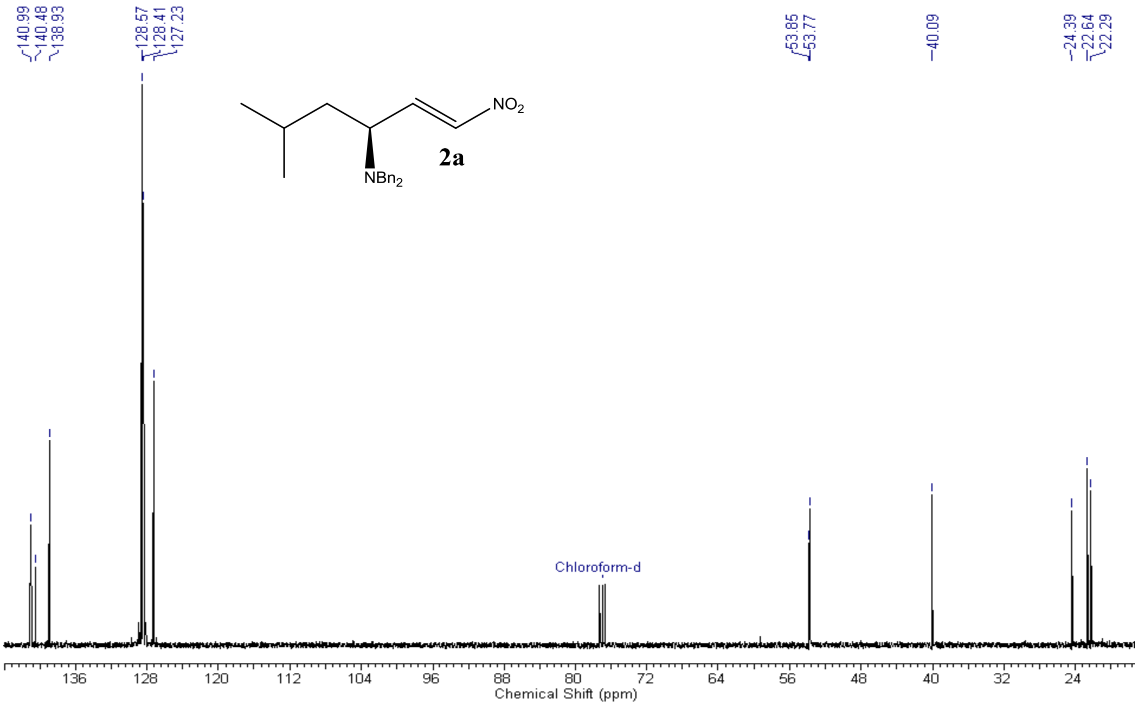

Spectrum 4: ^{13}C NMR (100 MHz, $CDCl_3$) of 2a

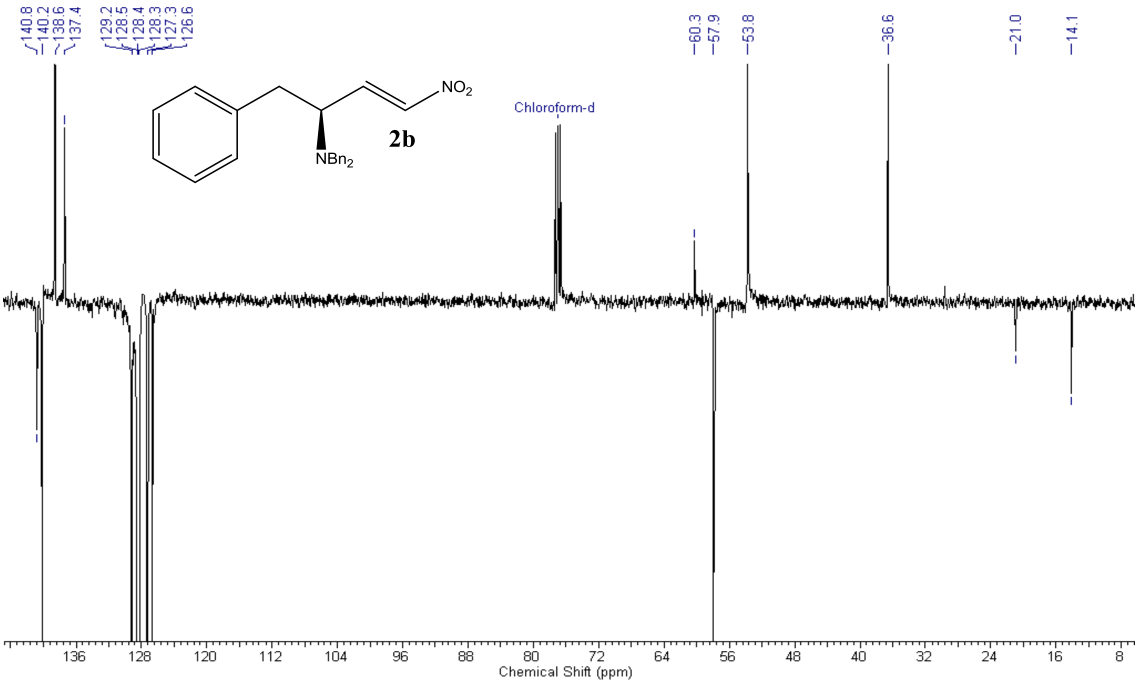

Spectrum 5: APT NMR (50 MHz, $CDCl_3$) of 2b

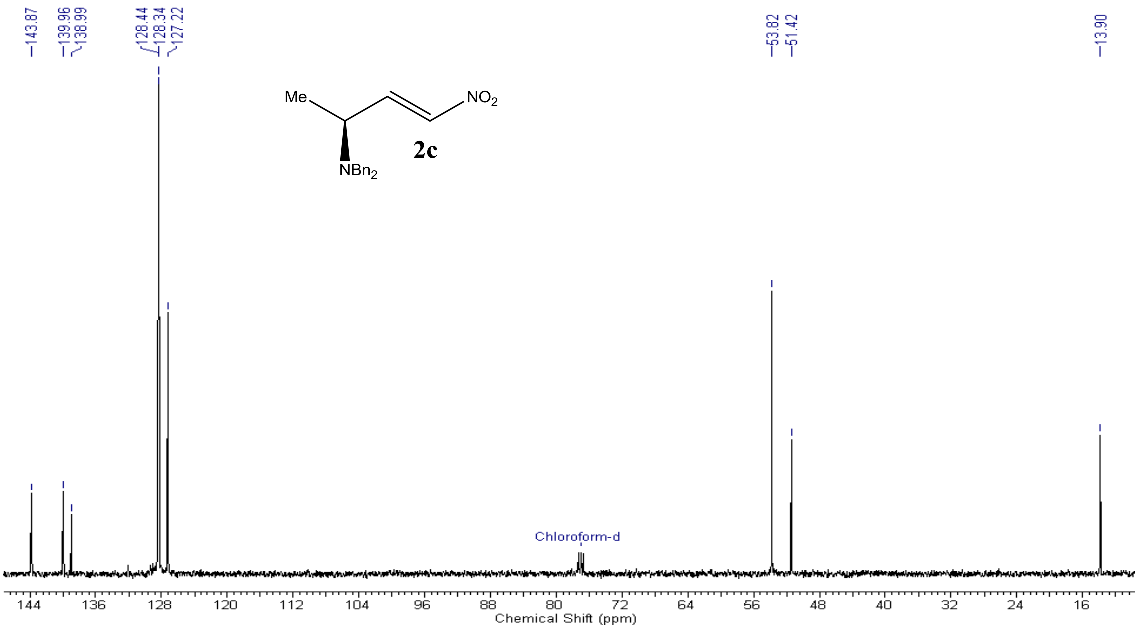

Spectrum 6: ^{13}C NMR (100 MHz, $CDCl_3$) of 2c

Mass Spectrum SmartFormula Report

**Analysis Info**		Acquisition Date	5/22/2012 4:28:50 PM
Analysis Name	D:\Data\Usuarios\Vera Patrocinio\ER202_Pos_22-05-12.d		
Method	Tune_pos_50-3000_Lab-Mass.m	Operator	BDAL@DE
Sample Name	ER202_Pos_22-05-12	Instrument / Ser#	micrOTOF 10368
Comment			

Acquisition Parameter

Source Type	ESI	Ion Polarity	Positive	Set Nebulizer	0.4 Bar
Focus	Not active			Set Dry Heater	180 °C
Scan Begin	50 m/z	Set Capillary	4500 V	Set Dry Gas	4.0 l/min
Scan End	3000 m/z	Set End Plate Offset	-500 V	Set Divert Valve	Waste

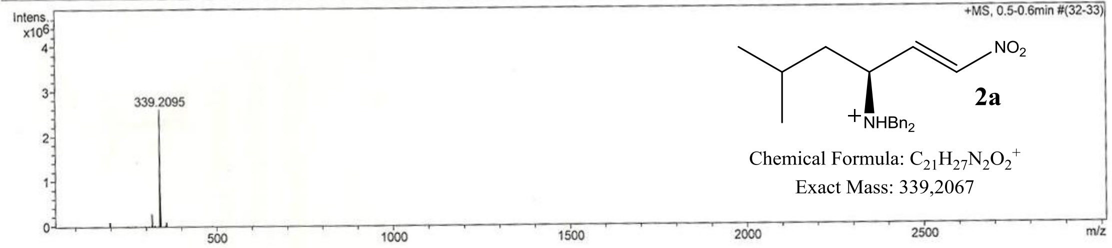

Meas. m/z	#	Formula	Score	m/z	err [mDa]	err [ppm]	mSigma	rdb	e⁻ Conf	N-Rule
339.2095	1	C 26 H 27	100.00	339.2107	1.3	3.7	8.1	13.5	even	ok
	2	C 21 H 27 N 2 O 2	17.49	339.2067	-2.8	-8.1	35.4	9.5	even	ok

Spectrum 7: HRMS of 2a

Mass Spectrum SmartFormula Report

**Analysis Info**		Acquisition Date	5/22/2012 4:15:39 PM
Analysis Name	D:\Data\Usuarios\Vera Patrocinio\ER204_Pos_22-05-12.d		
Method	Tune_pos_50-3000_Lab-Mass.m	Operator	BDAL@DE
Sample Name	ER204_Pos_22-05-12	Instrument / Ser#	micrOTOF 10368
Comment			

Acquisition Parameter

Source Type	ESI	Ion Polarity	Positive	Set Nebulizer	0.4 Bar
Focus	Not active			Set Dry Heater	180 °C
Scan Begin	50 m/z	Set Capillary	4500 V	Set Dry Gas	4.0 l/min
Scan End	3000 m/z	Set End Plate Offset	-500 V	Set Divert Valve	Waste

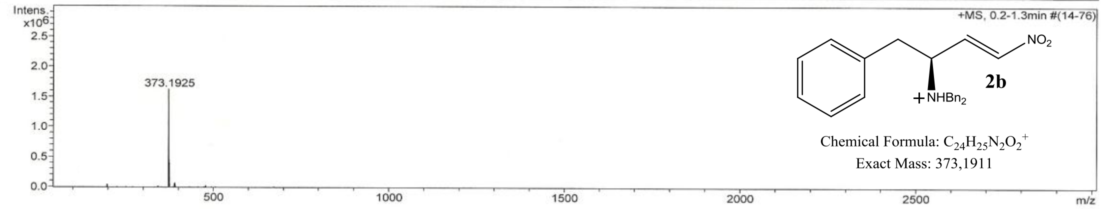

Meas. m/z	#	Formula	Score	m/z	err [mDa]	err [ppm]	mSigma	rdb	e⁻ Conf	N-Rule
373.1925	1	C 29 H 25	39.01	373.1951	2.6	7.0	12.7	17.5	even	ok
	2	C 24 H 25 N 2 O 2	100.00	373.1911	-1.4	-3.7	12.8	13.5	even	ok

Bruker Compass DataAnalysis 4.0 printed: 5/24/2012 10:47:16 AM Page 1 of 1

Spectrum 8: HRMS of 2b

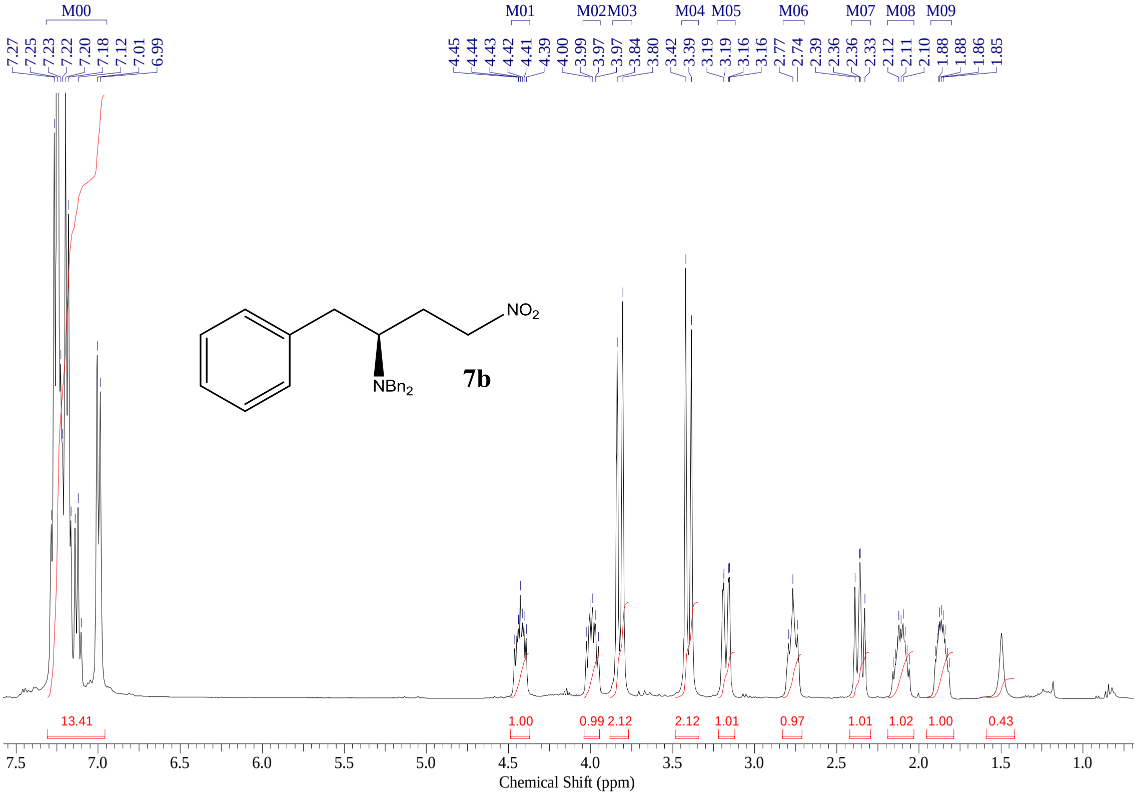

Spectrum 9: ^{1}H NMR (400 MHz, $CDCl_3$) of 7b.

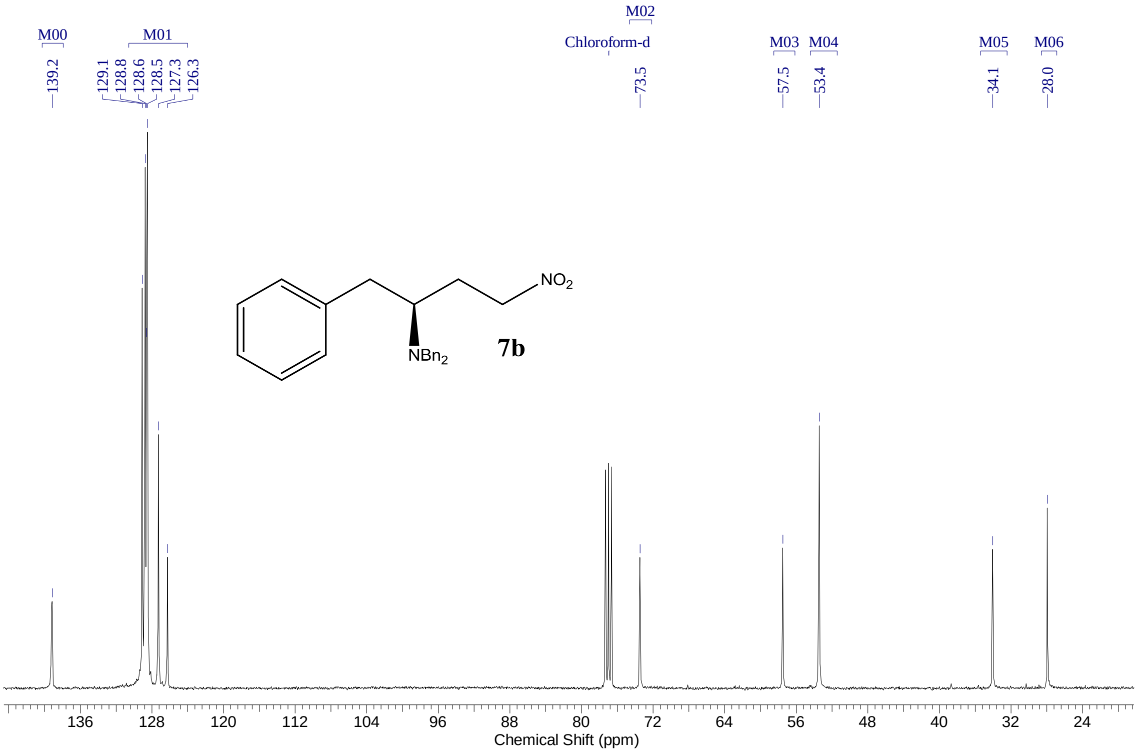

Spectrum 10: ^{13}C NMR (100 MHz, $CDCl_3$) of 7b

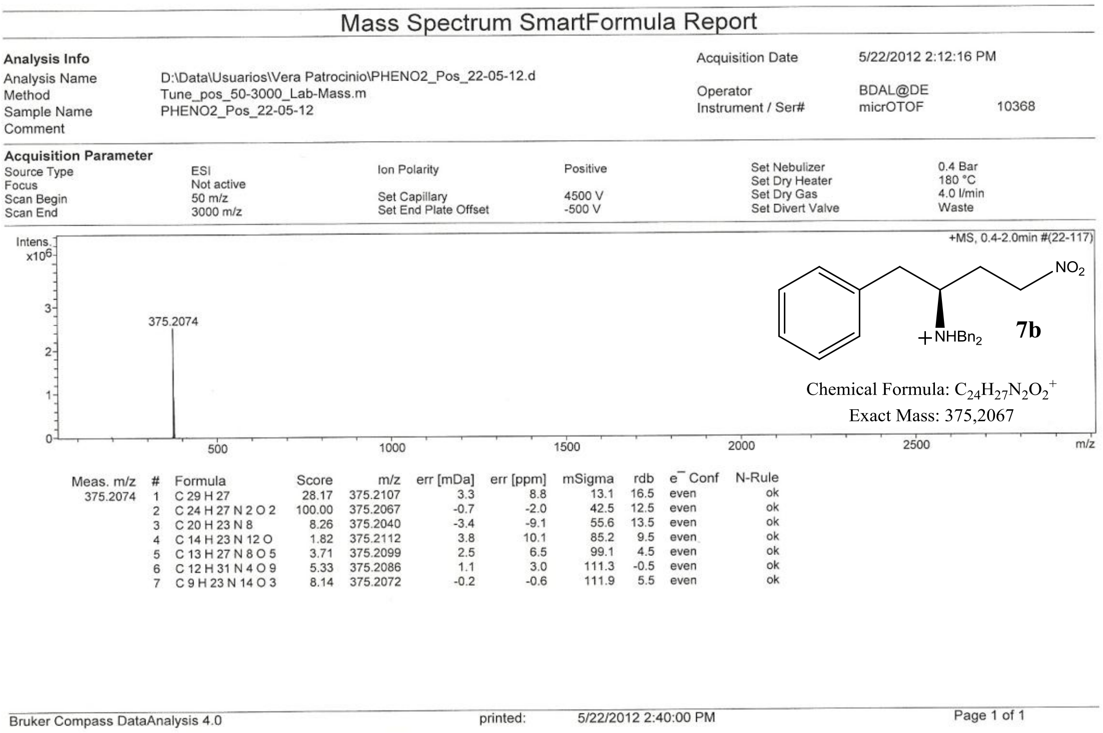

Mass Spectrum SmartFormula Report

**Analysis Info**		Acquisition Date	5/22/2012 2:12:16 PM
Analysis Name	D:\Data\Usuarios\Vera Patrocinio\PHENO2_Pos_22-05-12.d		
Method	Tune_pos_50-3000_Lab-Mass.m	Operator	BDAL@DE
Sample Name	PHENO2_Pos_22-05-12	Instrument / Ser#	micrOTOF 10368
Comment			

**Acquisition Parameter**					
Source Type	ESI	Ion Polarity	Positive	Set Nebulizer	0.4 Bar
Focus	Not active			Set Dry Heater	180 °C
Scan Begin	50 m/z	Set Capillary	4500 V	Set Dry Gas	4.0 l/min
Scan End	3000 m/z	Set End Plate Offset	-500 V	Set Divert Valve	Waste

Meas. m/z	#	Formula	Score	m/z	err [mDa]	err [ppm]	mSigma	rdb	e⁻ Conf	N-Rule
375.2074	1	C 29 H 27	28.17	375.2107	3.3	8.8	13.1	16.5	even	ok
	2	C 24 H 27 N 2 O 2	100.00	375.2067	-0.7	-2.0	42.5	12.5	even	ok
	3	C 20 H 23 N 8	8.26	375.2040	-3.4	-9.1	55.6	13.5	even	ok
	4	C 14 H 23 N 12 O	1.82	375.2112	3.8	10.1	85.2	9.5	even	ok
	5	C 13 H 27 N 8 O 5	3.71	375.2099	2.5	6.5	99.1	4.5	even	ok
	6	C 12 H 31 N 4 O 9	5.33	375.2086	1.1	3.0	111.3	-0.5	even	ok
	7	C 9 H 23 N 14 O 3	8.14	375.2072	-0.2	-0.6	111.9	5.5	even	ok

Bruker Compass DataAnalysis 4.0 printed: 5/22/2012 2:40:00 PM Page 1 of 1

Spectrum 11: HRMS of 7b

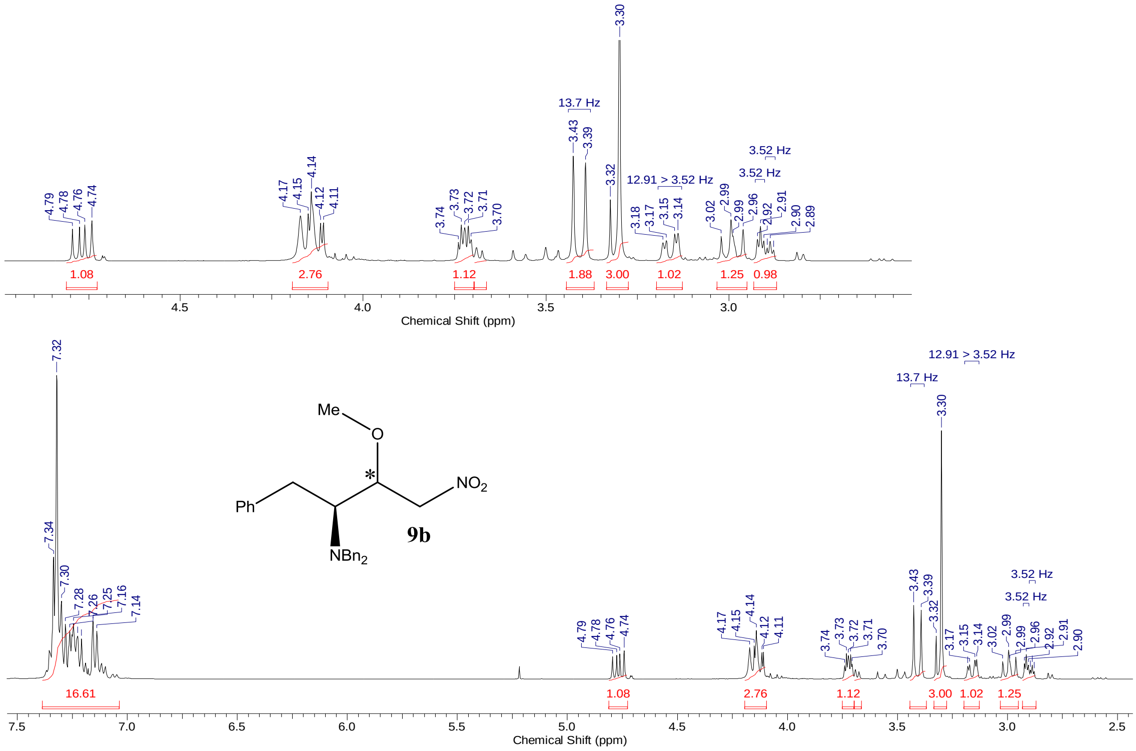

Spectrum 12: ^{1}H NMR (400 MHz, $CDCl_3$) of 9b

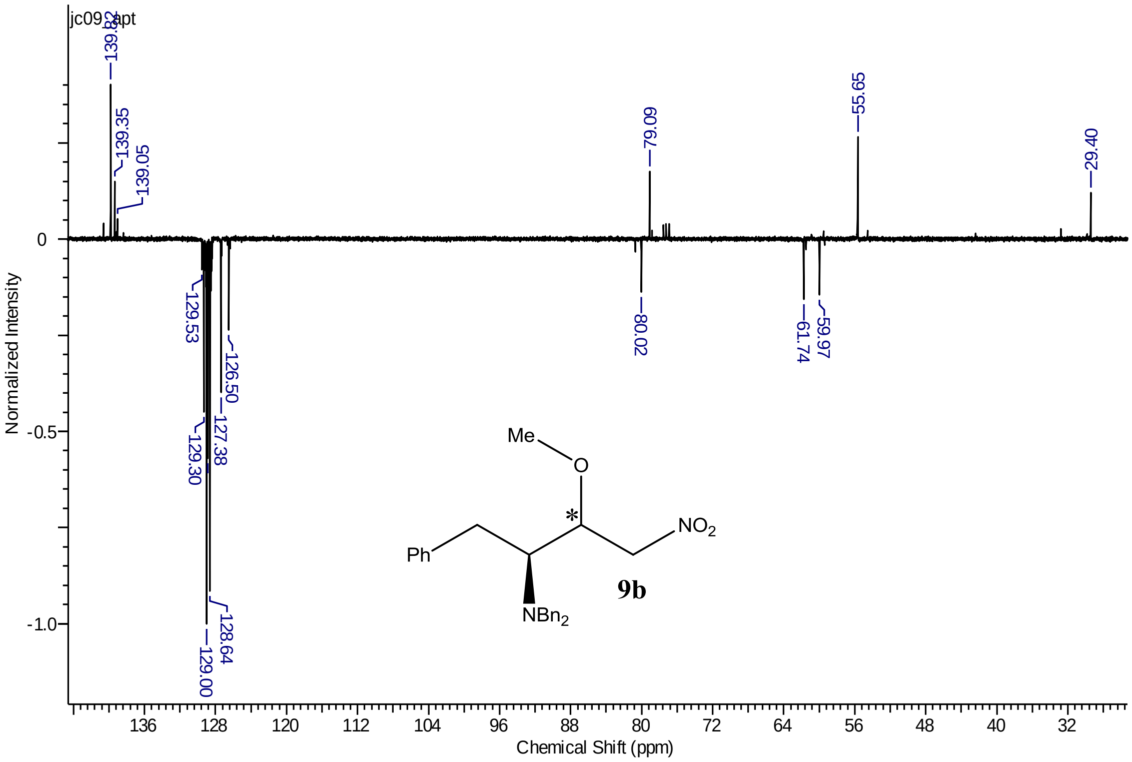

Spectrum 13: ^{13}C NMR (100 MHz, $CDCl_3$) (APT) of 9b

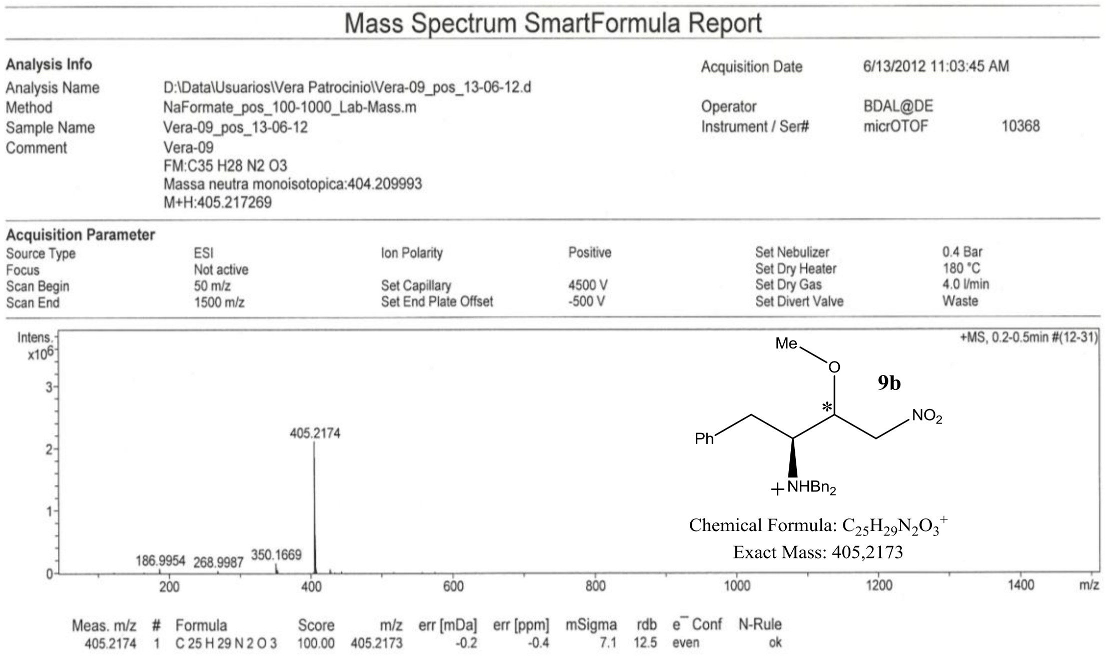

Mass Spectrum SmartFormula Report

**Analysis Info**		Acquisition Date	6/13/2012 11:03:45 AM
Analysis Name	D:\Data\Usuarios\Vera Patrocinio\Vera-09_pos_13-06-12.d		
Method	NaFormate_pos_100-1000_Lab-Mass.m	Operator	BDAL@DE
Sample Name	Vera-09_pos_13-06-12	Instrument / Ser#	micrOTOF 10368
Comment	Vera-09<br>FM:C35 H28 N2 O3<br>Massa neutra monoisotopica:404.209993<br>M+H:405.217269		

Acquisition Parameter

Source Type	ESI	Ion Polarity	Positive	Set Nebulizer	0.4 Bar
Focus	Not active			Set Dry Heater	180 °C
Scan Begin	50 m/z	Set Capillary	4500 V	Set Dry Gas	4.0 l/min
Scan End	1500 m/z	Set End Plate Offset	-500 V	Set Divert Valve	Waste

Meas. m/z	#	Formula	Score	m/z	err [mDa]	err [ppm]	mSigma	rdb	e⁻ Conf	N-Rule
405.2174	1	C 25 H 29 N 2 O 3	100.00	405.2173	-0.2	-0.4	7.1	12.5	even	ok

Spectrum 14: HRMS of 9b

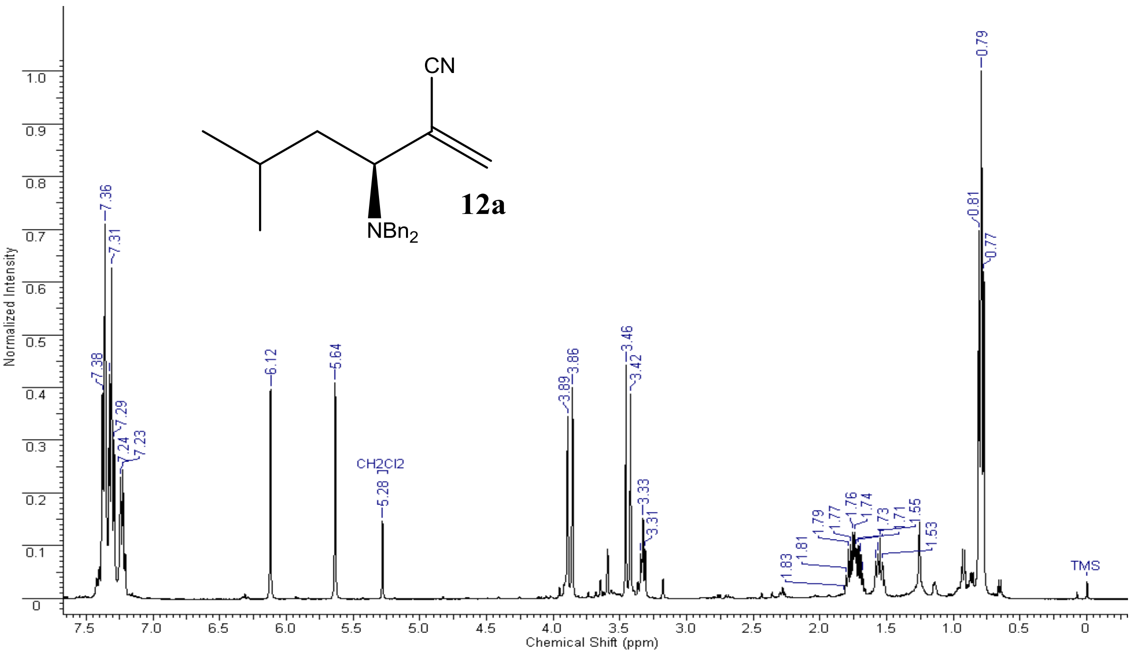

Spectrum 15: ^{1}H NMR (400 MHz, $CDCl_3$) of 12a

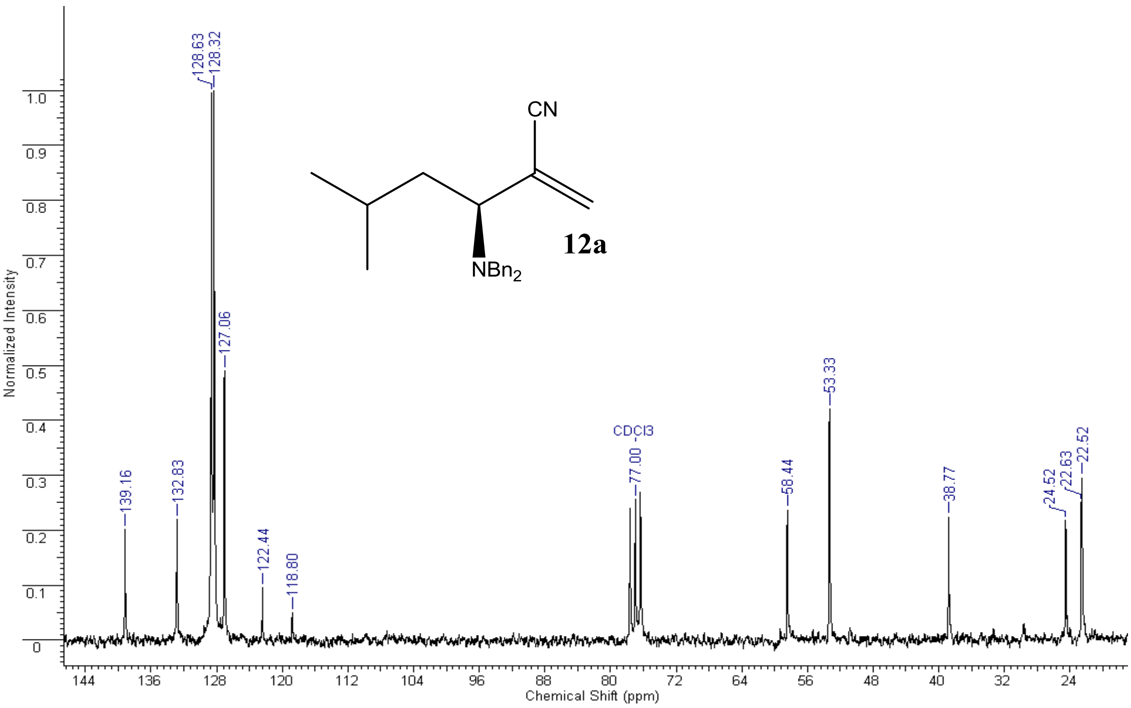

Spectrum 16: **^{13}C NMR (100 MHz, $CDCl_3$) of 12a**

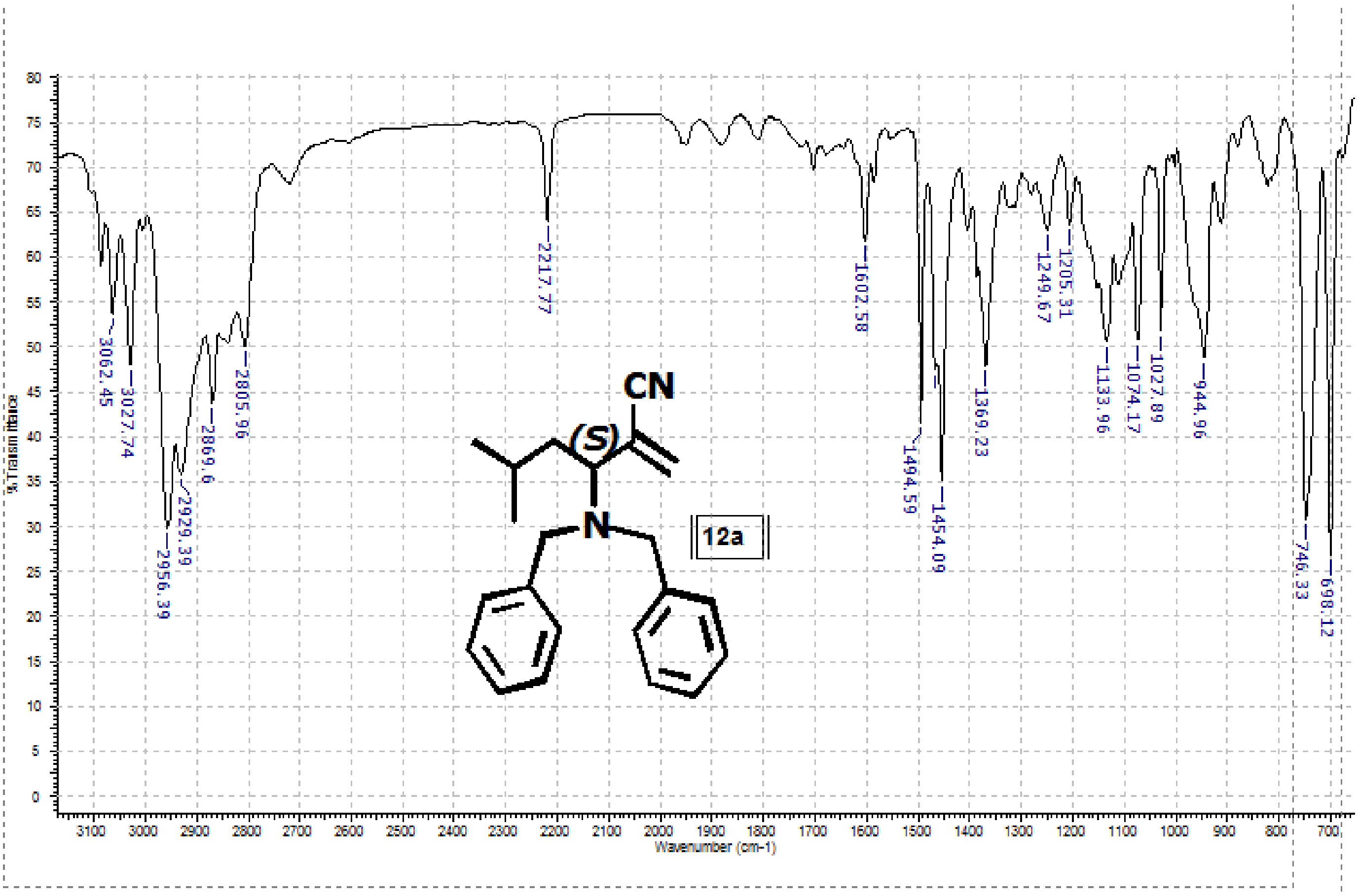

Specrtrum 17: IR of 12a

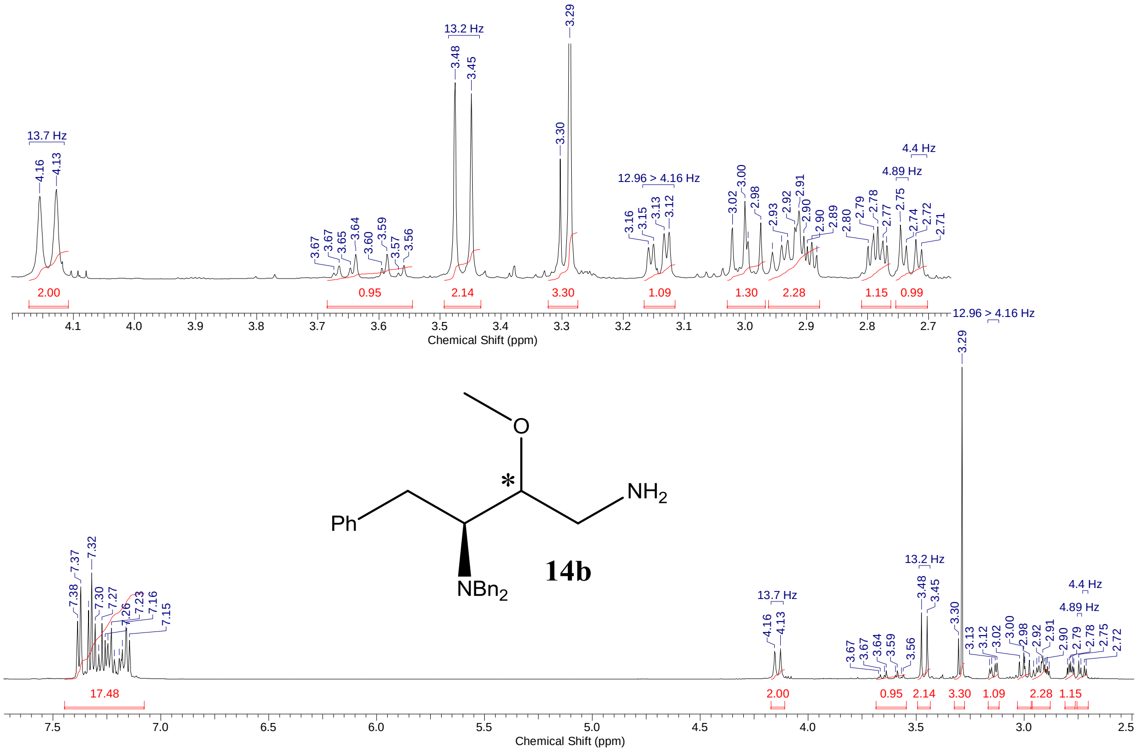

Spectrum 18: ^{1}H NMR (400 MHz, $CDCl_3$) of 14b

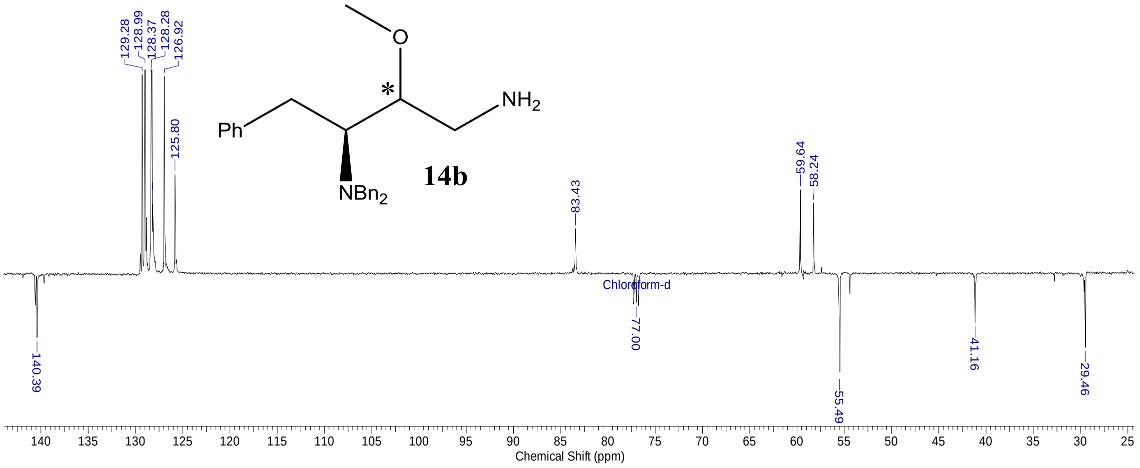

Spectrum 19: ^{13}C NMR-APT (100 MHz, $CDCl_3$) of 14b

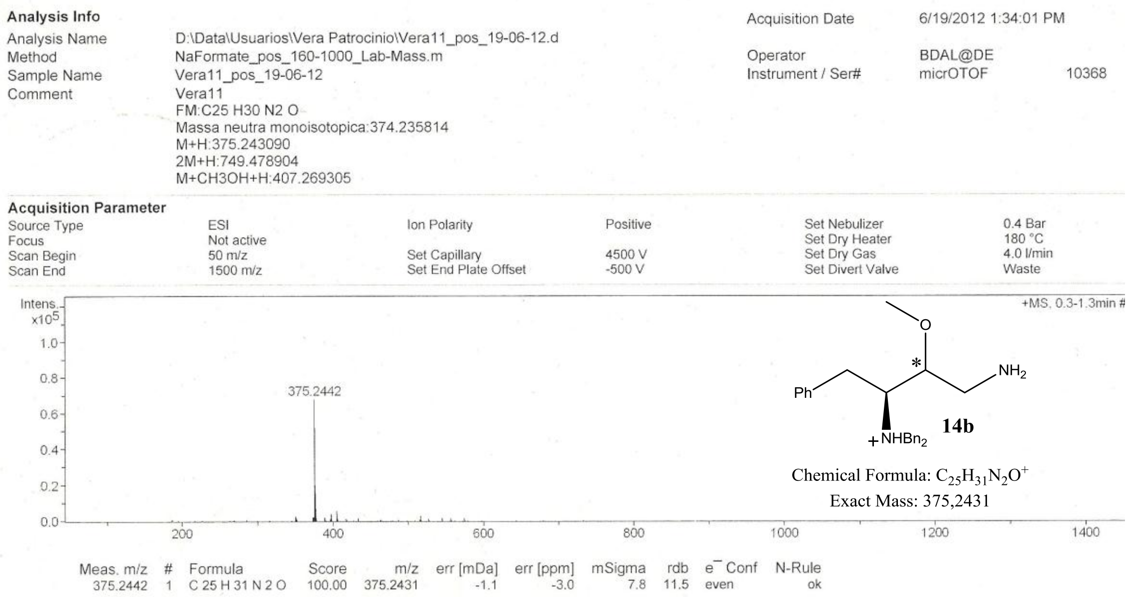

Spectrum 20: HRMS of 14b

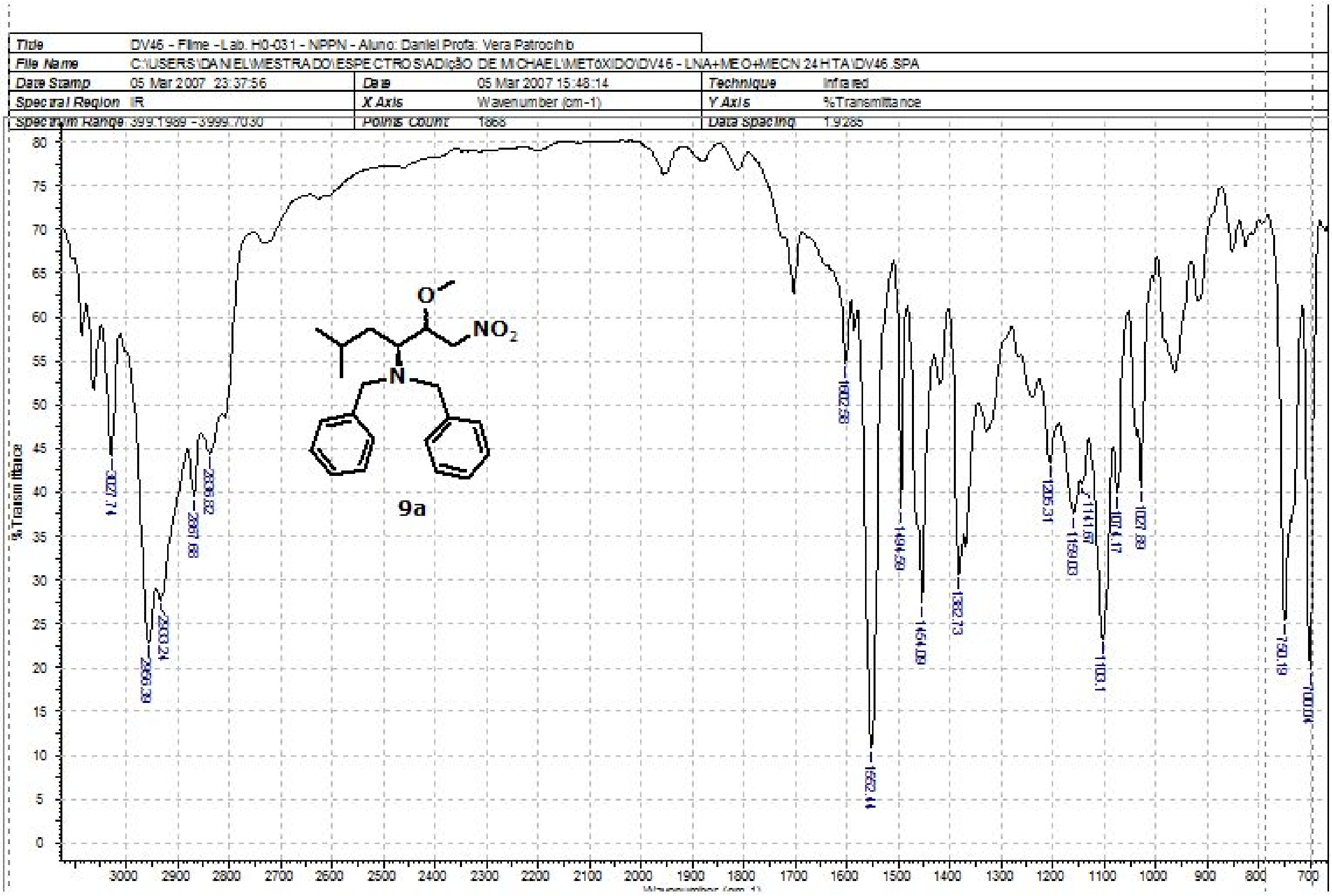

Spectrum 21: **IR of 9a**

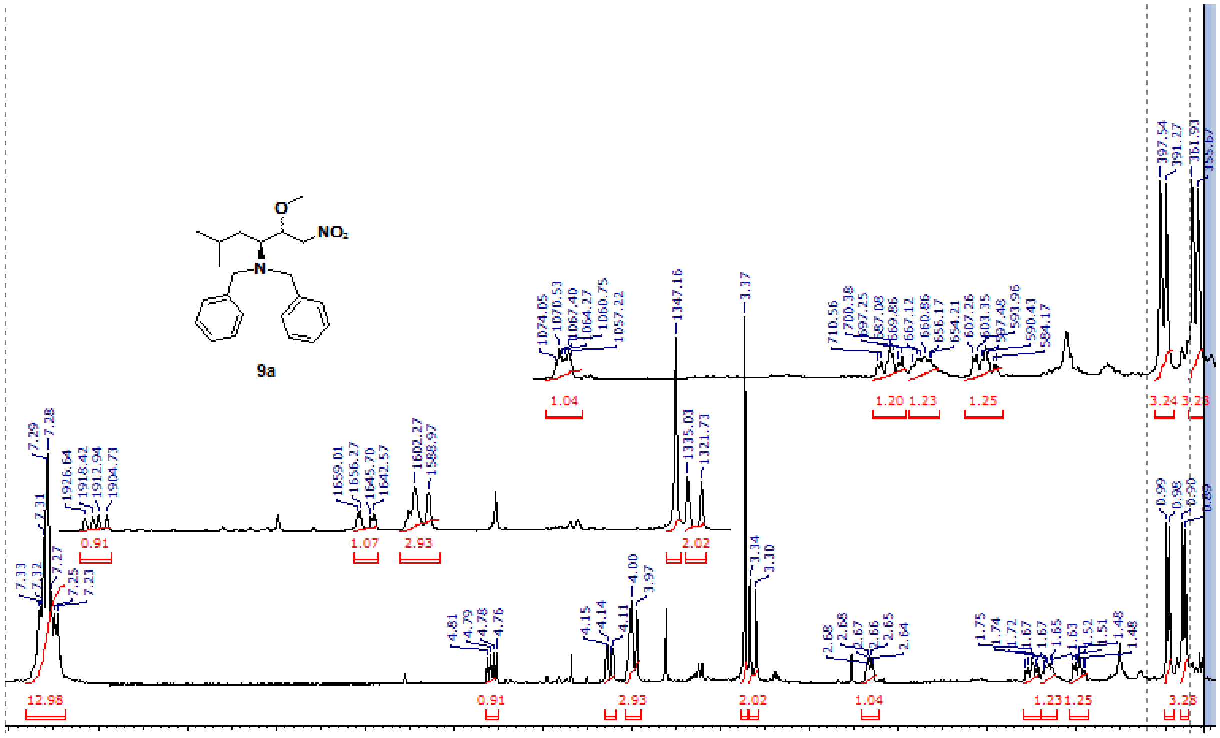

Spectrum 22: ^{1}H NMR of 9a (400 MHz - $CDCl_3$) - diastereomeric mixture 53:47

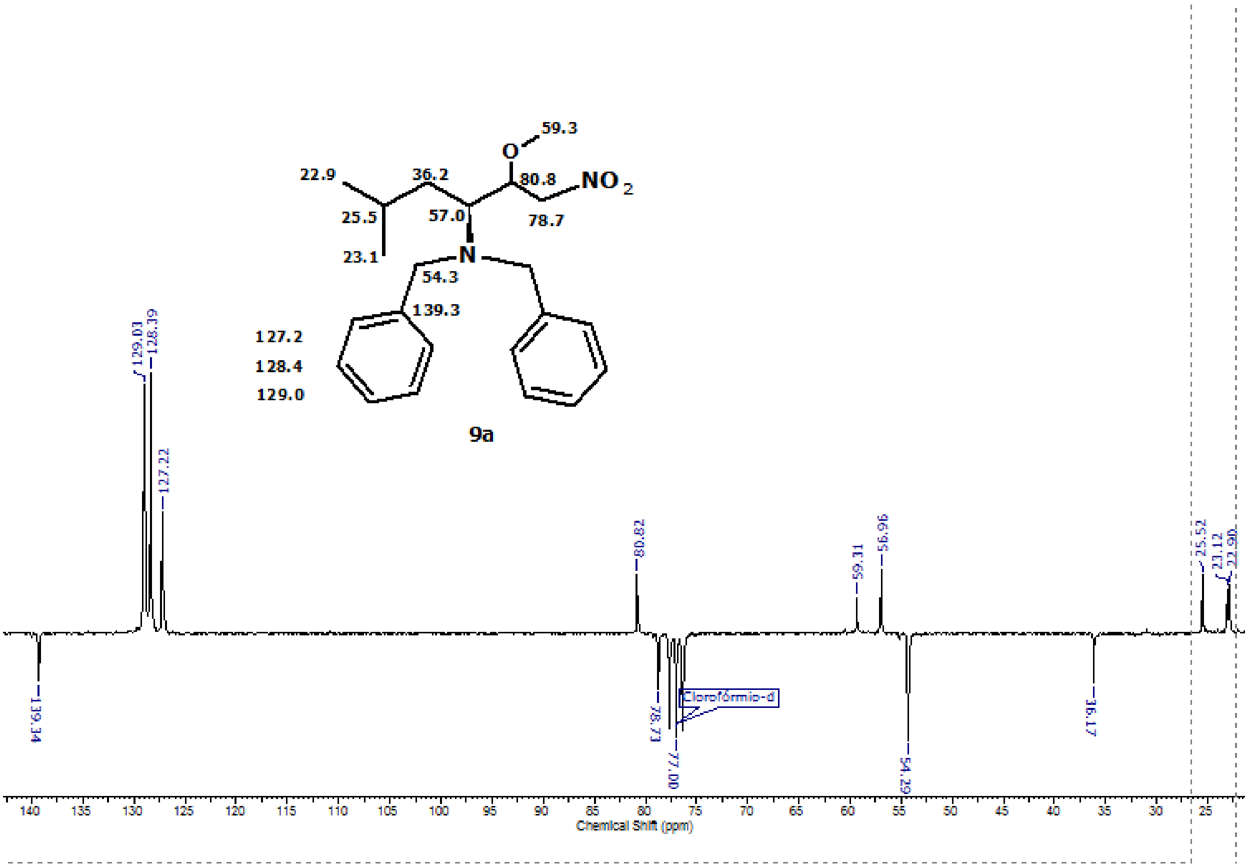

Spectrum 23: ^{13}C NMR-APT (400 MHz, $CDCl_3$) of 9a (d.e. >95%)

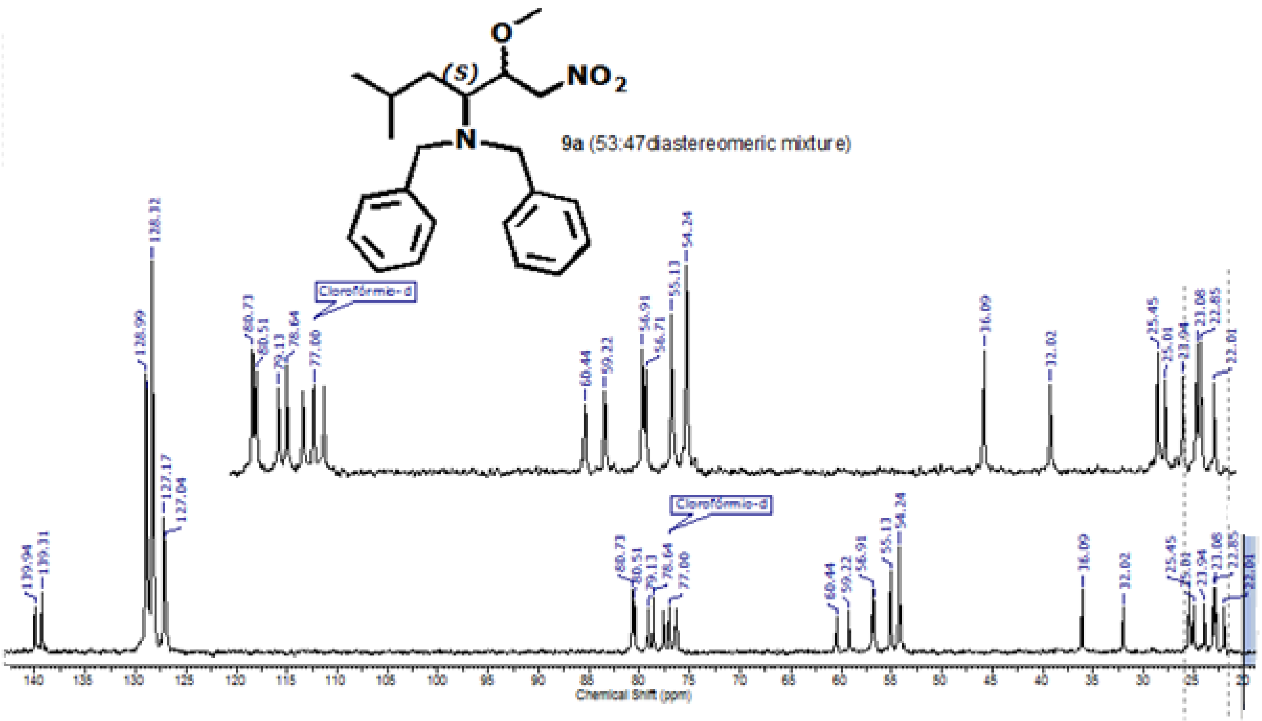

Spectrum 24: ^{13}C NMR of 9a (50 MHz, $CDCl_3$) (53:47 diastereomeric mixture)

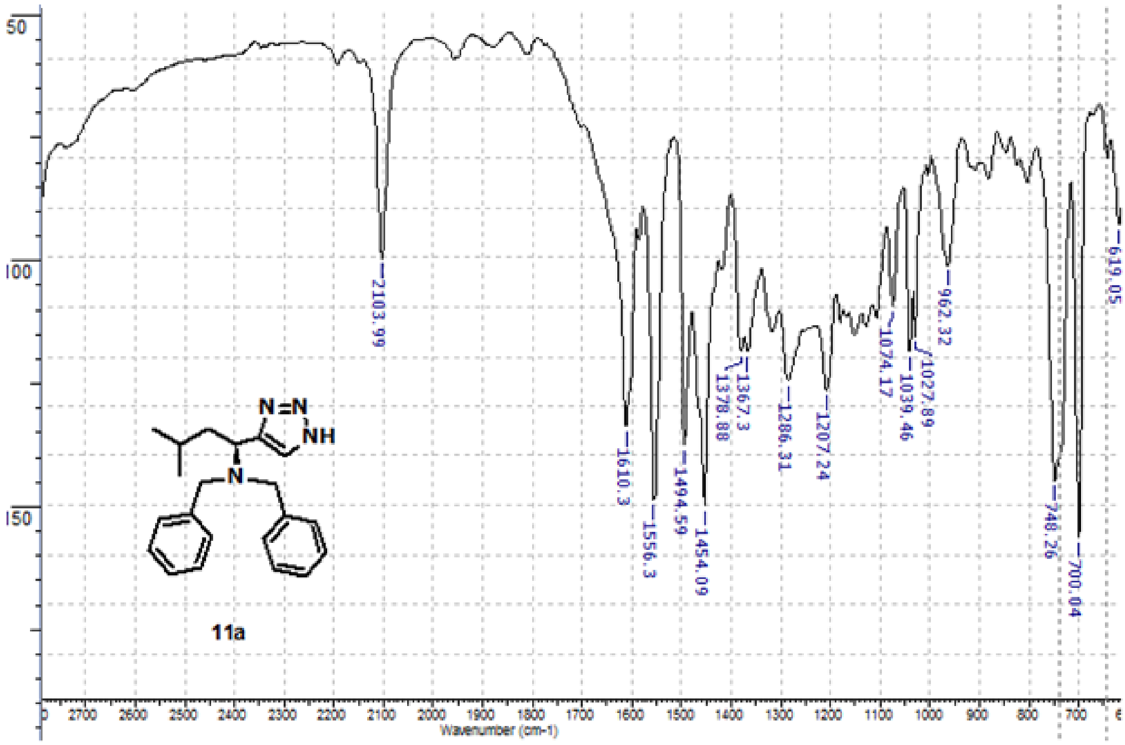

Spectrum 25: IR of 11a

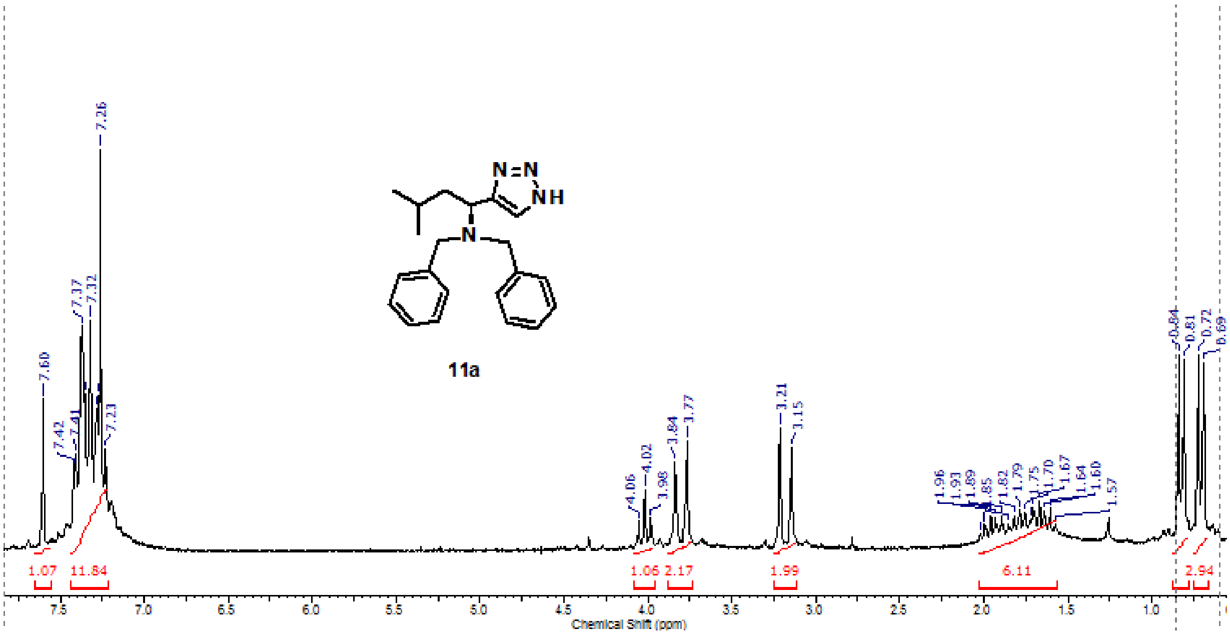

Spectrum 26: ^{1}H NMR (400 MHz, $CDCl_3$) of 11a (Mixture of tautomers)

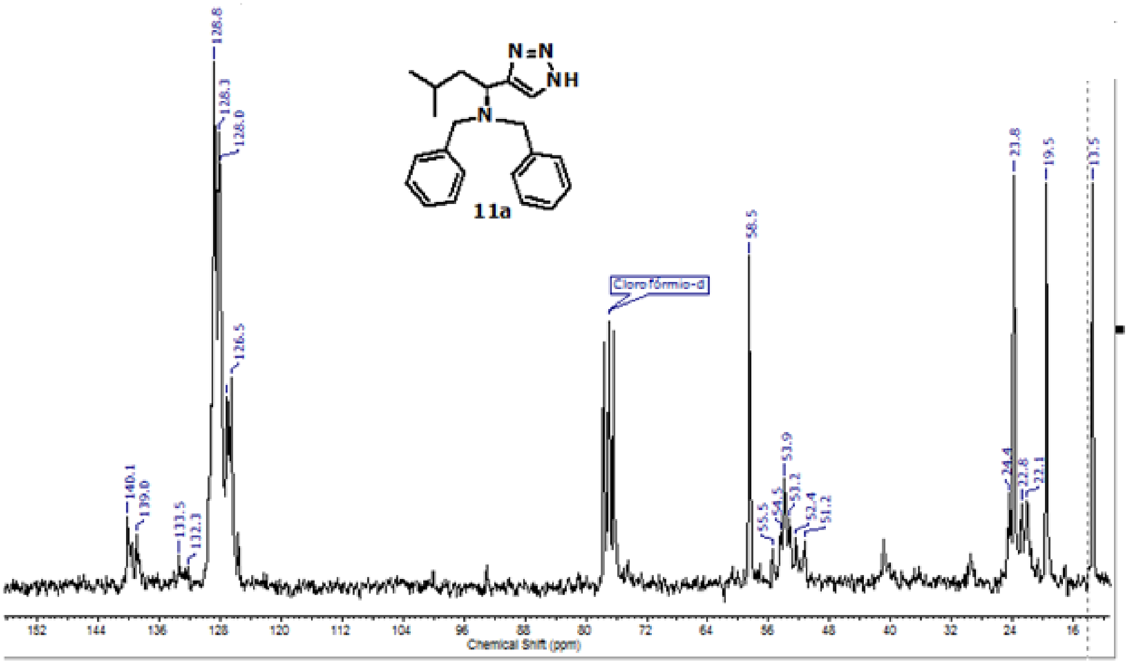

Spectrum 27: ^{13}C NMR (50 MHz, $CDCl_3$) of 11a (Mixture of tautomers)

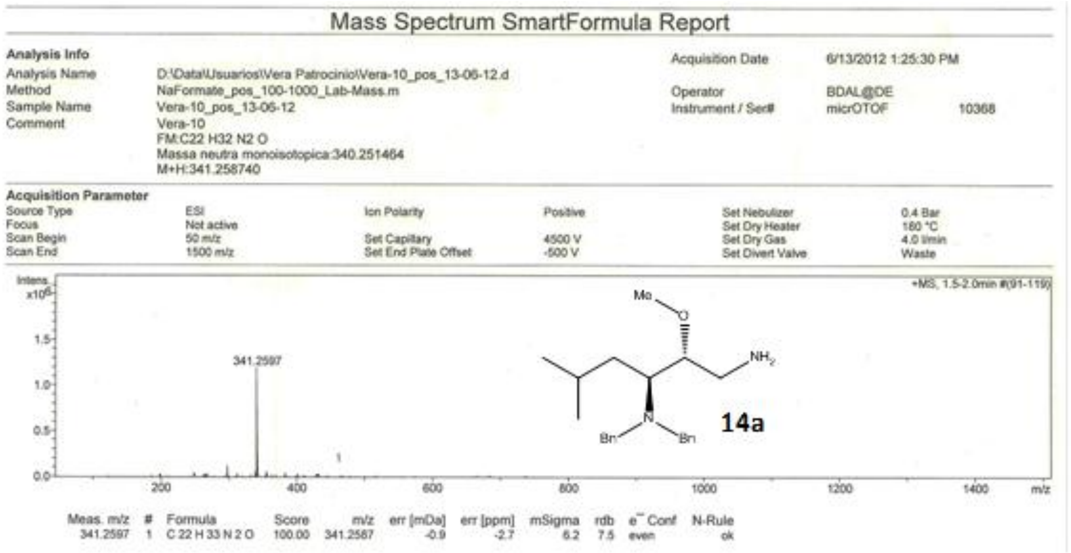

Mass Spectrum SmartFormula Report

Analysis Info

Analysis Name	D:\Data\Usuarios\Vera Patrocinio\Vera-10_pos_13-06-12.d	Acquisition Date	6/13/2012 1:25:30 PM
Method	NaFormate_pos_100-1000_Lab-Mass.m	Operator	BDAL@DE
Sample Name	Vera-10_pos_13-06-12	Instrument / Ser#	micrOTOF 10368
Comment	Vera-10<br>FM:C22 H32 N2 O<br>Massa neutra monoisotopica:340.251464<br>M+H:341.258740		

Acquisition Parameter

Source Type	ESI	Ion Polarity	Positive	Set Nebulizer	0.4 Bar
Focus	Not active			Set Dry Heater	180 °C
Scan Begin	50 m/z	Set Capillary	4500 V	Set Dry Gas	4.0 l/min
Scan End	1500 m/z	Set End Plate Offset	-500 V	Set Divert Valve	Waste

Meas. m/z	#	Formula	Score	m/z	err [mDa]	err [ppm]	mSigma	rdb	e⁻ Conf	N-Rule
341.2597	1	C 22 H 33 N 2 O	100.00	341.2587	-0.9	-2.7	6.2	7.5	even	ok

Spectrum 28: HRMS of 14a

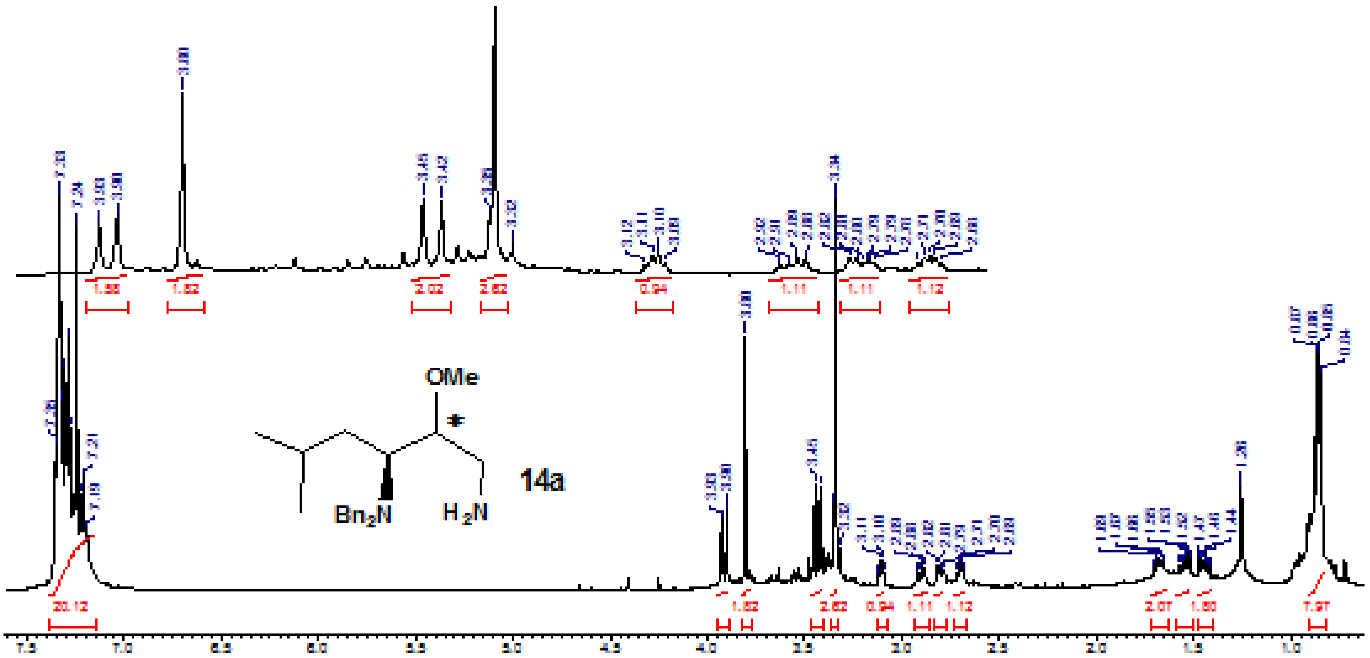

Spectrum 29: ^{13}C NMR of 14a (400 MHz, CD_3OD)

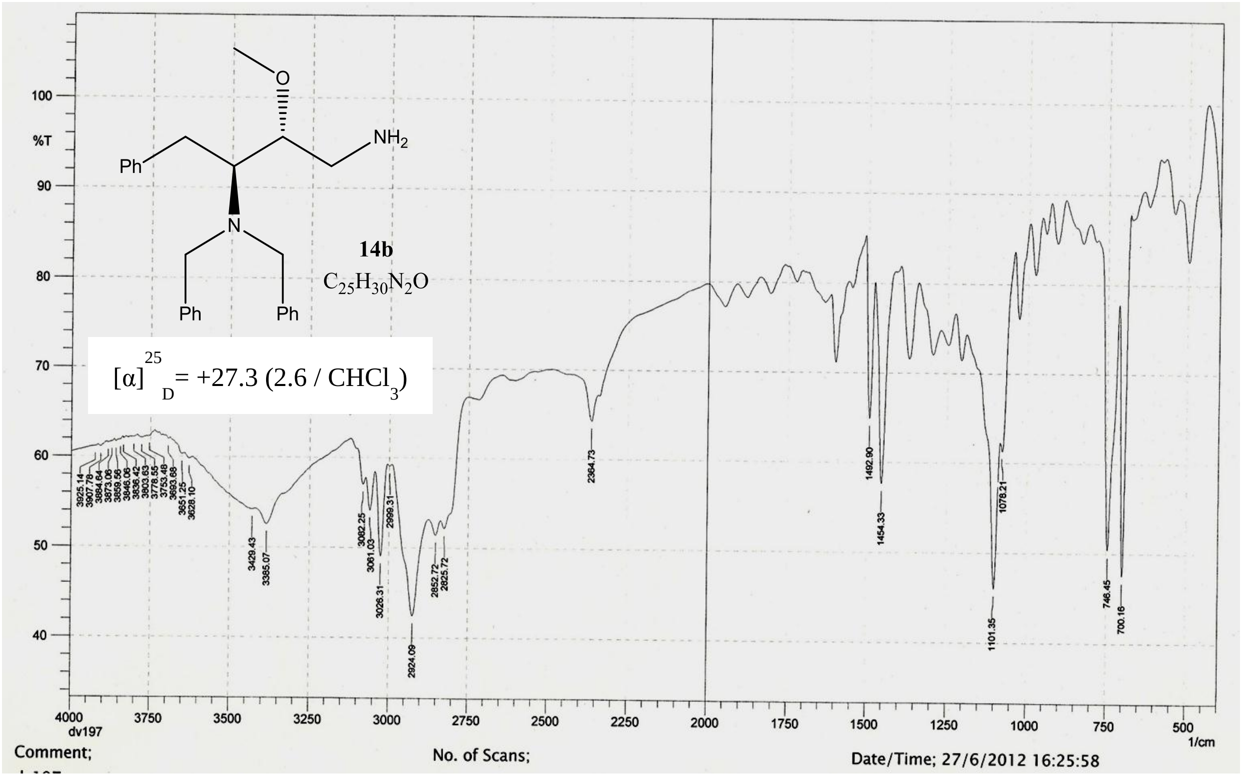

Spectrum 30: IR of 14b

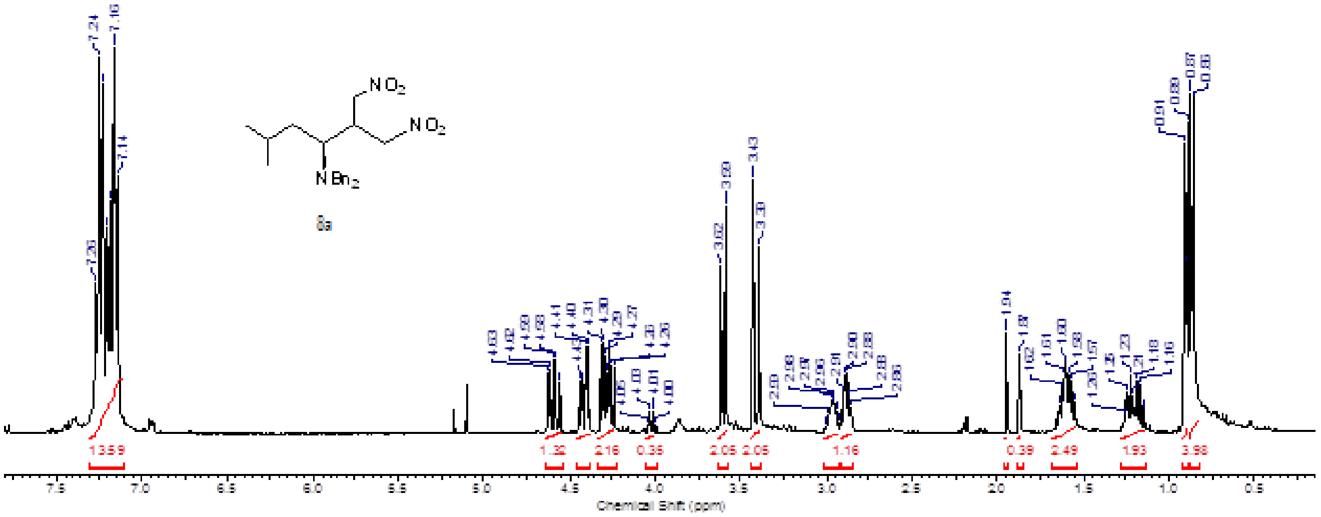

Spectrum 31: ^1H NMR (400 MHz, $CDCl_3$) of 8a

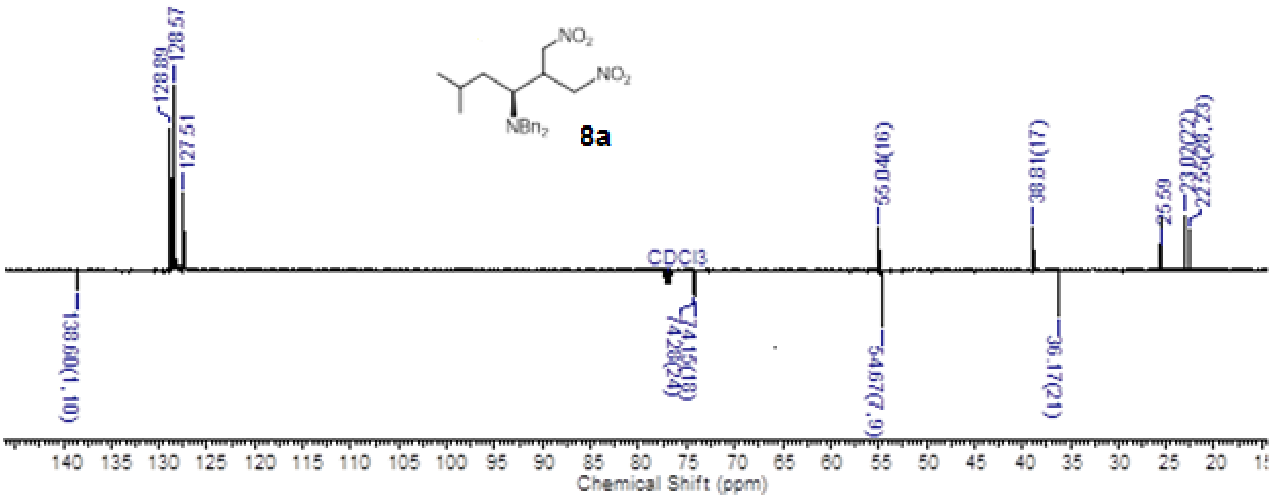

Spectrum 32: ^{13}C NMR (50 MHz, $CDCl_3$) of 8a

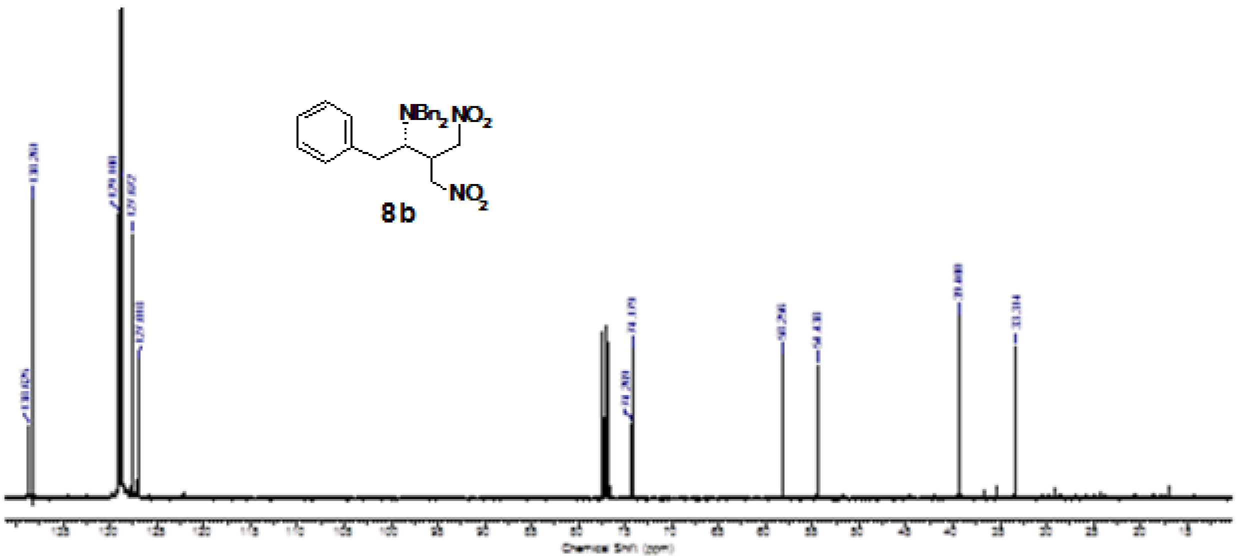

Spectrum 33: ^{13}C NMR (50 MHz, $CDCl_3$) of 8b

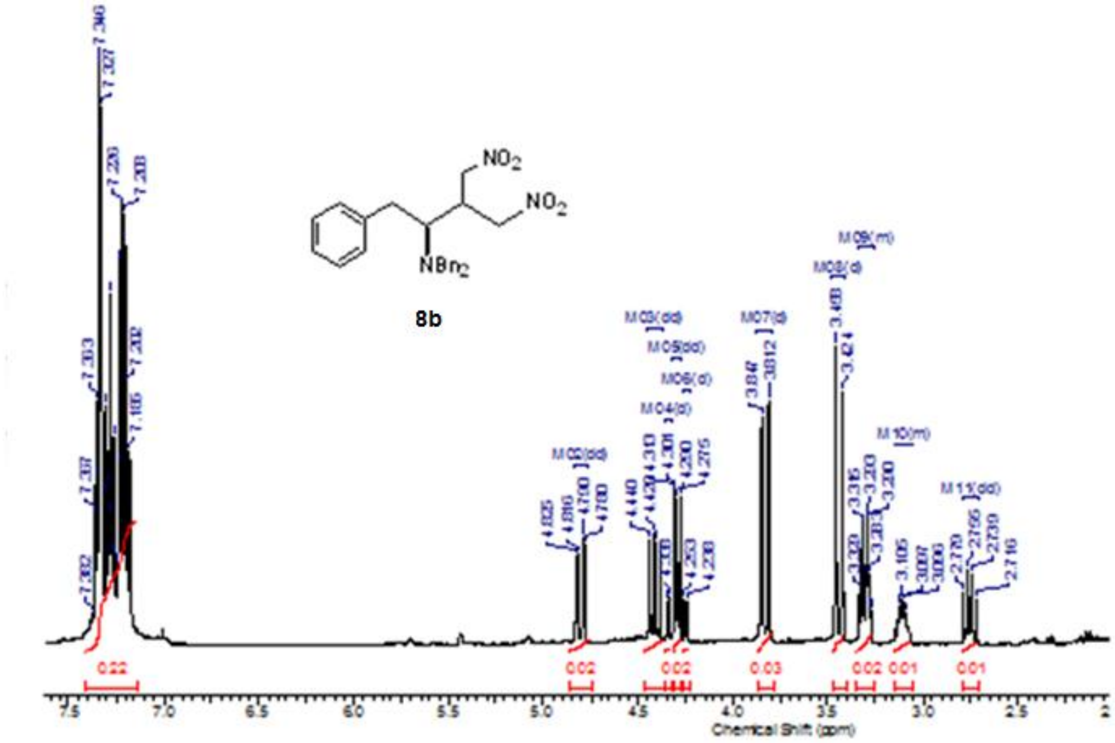

Spectrum 34: ^{1}H NMR (400 MHz, $CDCl_3$) of 8b.

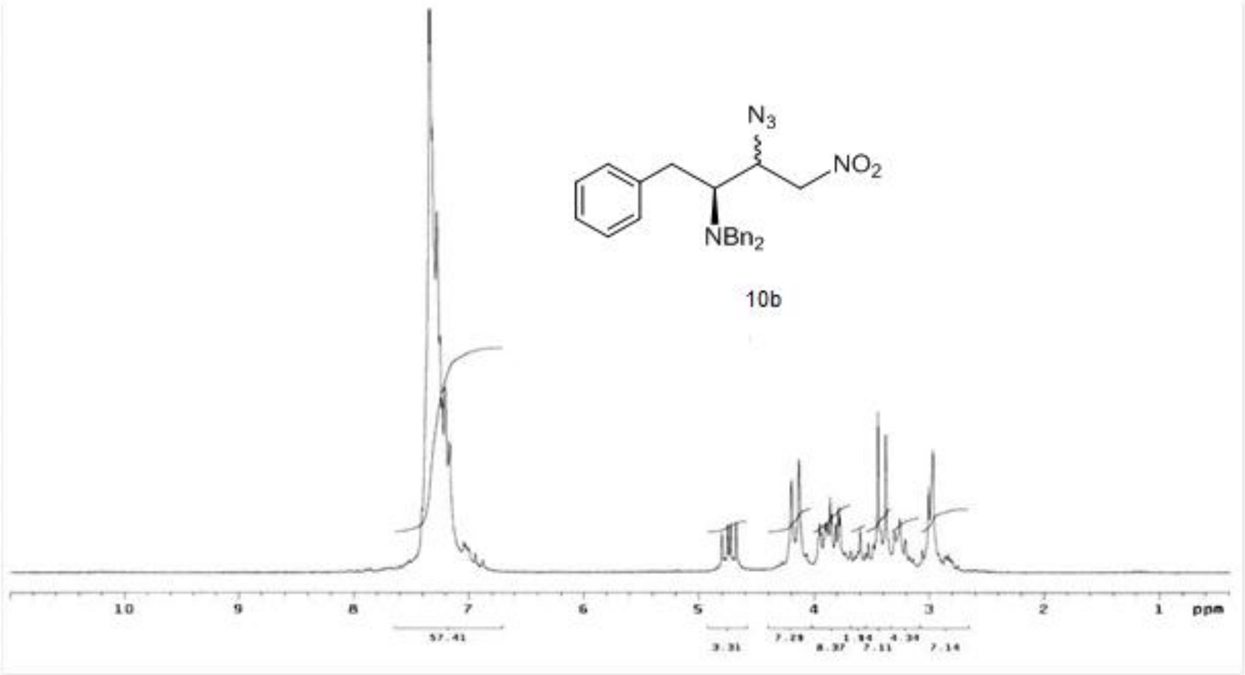

Spectrum 35: ^{1}H NMR (200 MHz, CDCl$_3$) of 10b.

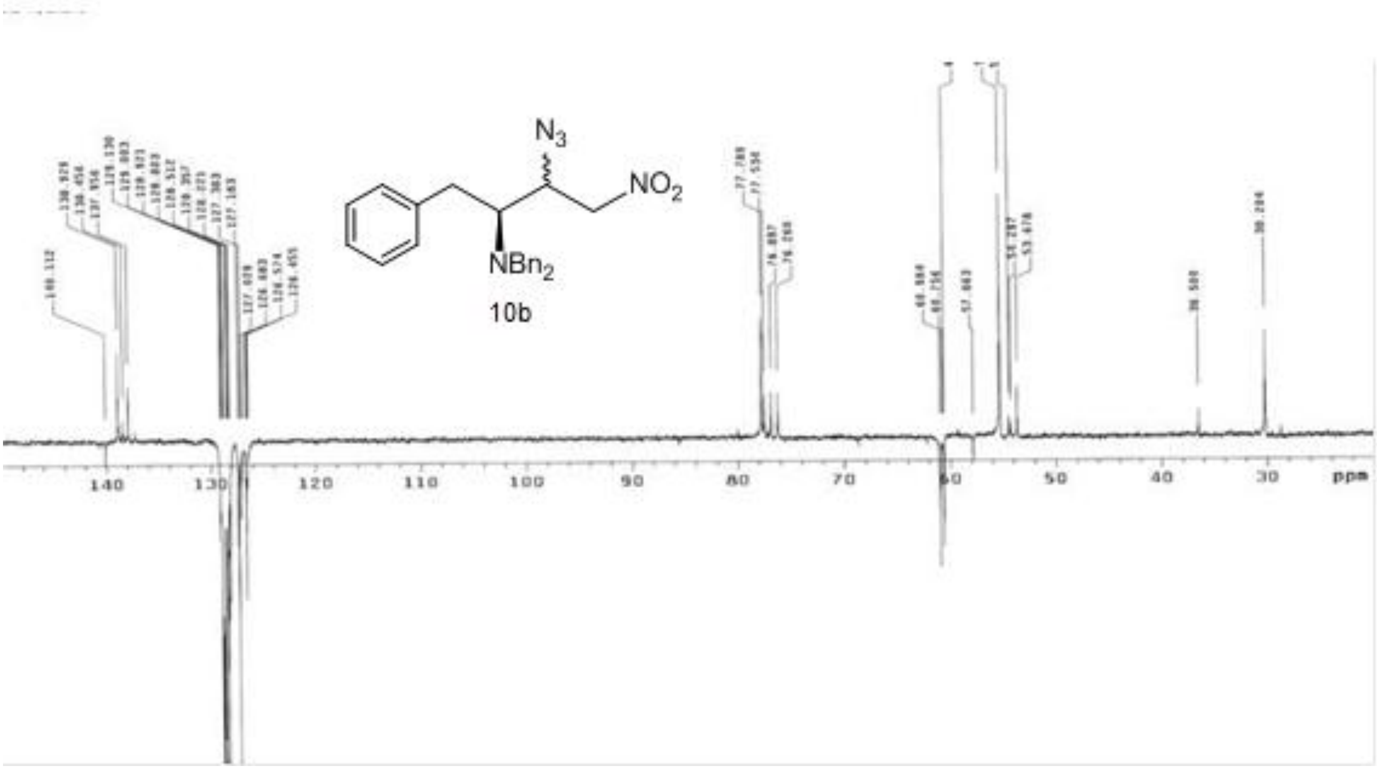

Spectrum 36: ^{13}C NMR-APT (200 MHz, CDCl$_3$) of 10b.
